# Production of rare ginsenosides by biotransformation of *Panax notoginseng* saponins using *Aspergillus fumigatus*

**DOI:** 10.1186/s40643-024-00794-0

**Published:** 2024-08-09

**Authors:** Lian Yang, Dongmei Lin, Feixing Li, Xiuming Cui, Dengji Lou, Xiaoyan Yang

**Affiliations:** 1 Faculty of Life Science and Technology, Kunming University of Science and Technology, Kunming, 650500 People’s Republic of China; 2 School of Chemical, Biological and Environmental Sciences, Yuxi Normal University, Yuxi, 653100 People’s Republic of China

**Keywords:** *Panax notoginseng* saponins, Biotransformation, Rare ginsenosides, *Aspergillus fumigatus*, Antimicrobial activity

## Abstract

*Panax notoginseng* saponins (PNS) are the main active components of *Panax notoginseng*. But after oral administration, they need to be converted into rare ginsenosides by human gut microbiota and gastric juice before they can be readily absorbed into the bloodstream and exert their effects. The sources of rare ginsenosides are extremely limited in *P. notoginseng* and other medical plants, which hinders their application in functional foods and drugs. Therefore, the production of rare ginsenosides by the transformation of PNS using *Aspergillus fumigatus* was studied in this research. During 50 days at 25 ℃ and 150 rpm, *A. fumigatus* transformed PNS to 14 products (**1**–**14**). They were isolated by varied chromatographic methods, such as silica gel column chromatography, Rp-C_18_ reversed phase column chromatography, semi-preparative HPLC, Sephadex LH-20 gel column chromatography, and elucidated on the basis of their ^1^H-NMR, ^13^C-NMR and ESIMS spectroscopic data. Then, the transformed products (**1**–**14**) were isolated and identified as Rk_3_, Rh_4_, 20 (*R*)-Rh_1_, 20 (*S*)-Protopanaxatriol, C-K, 20 (*R*)-Rg_3_, 20 (*S*)-Rg_3_, 20 (*S*)-Rg_2_, 20 (*R*)-R_2_, Rk_1_, Rg_5_, 20 (*S*)-R_2_, 20 (*R*)-Rg_2_, and 20 (*S*)-I_,_ respectively. In addition, all transformed products (**1**–**14**) were tested for their antimicrobial activity. Among them, compounds **5** (C-K) and **7** [20 (*S*)-Rg_3_] showed moderate antimicrobial activities against *Staphylococcus aureus* and *Candida albicans* with MIC values of 6.25, 1.25 μg/mL and 1.25, 25 μg/mL, respectively. This study lays the foundation for production of rare ginsenosides.

**Graphical abstract:**

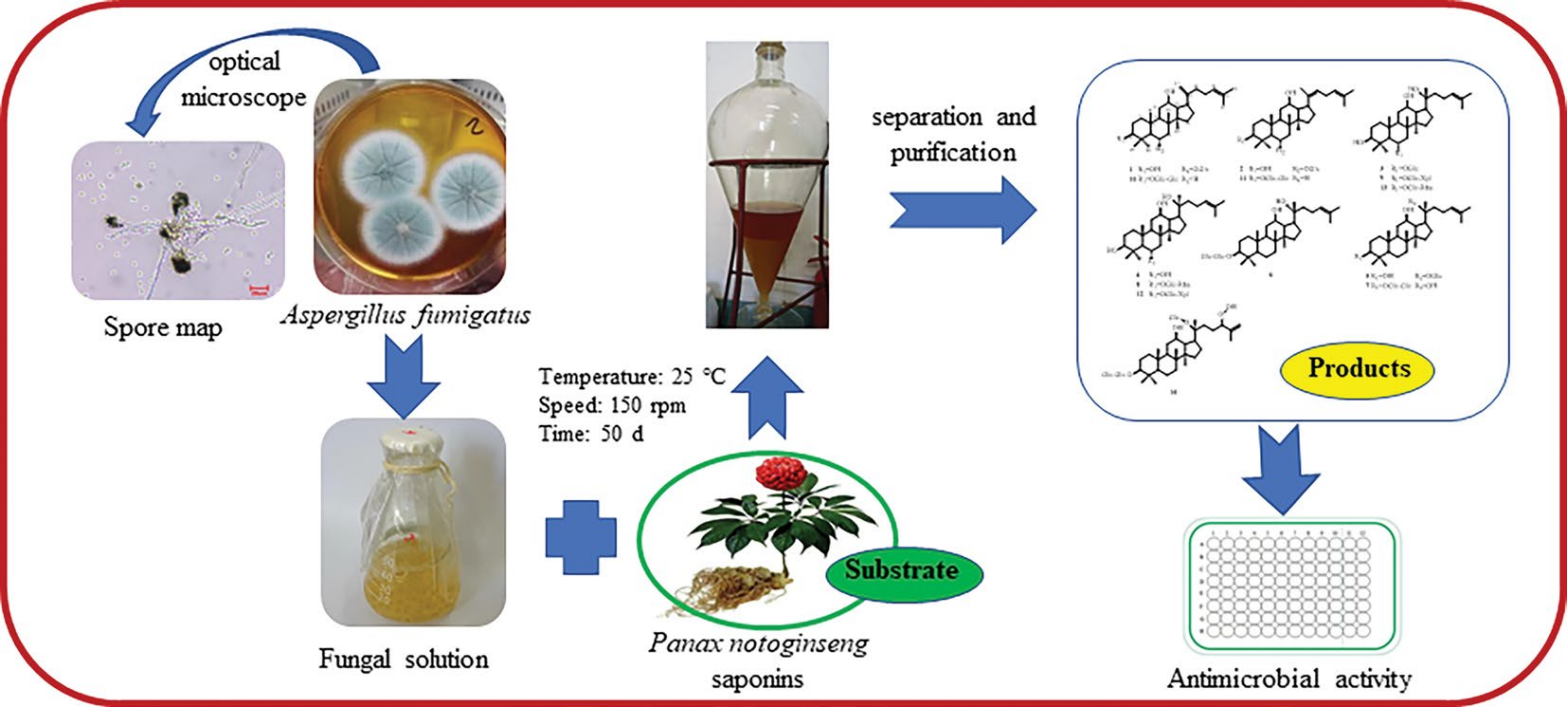

**Supplementary Information:**

The online version contains supplementary material available at 10.1186/s40643-024-00794-0.

## Introduction

*Panax notoginseng* (Burk.) F.H. Chen is a well-known traditional Chinese medicine, which has various biological activities (Li et al. 2022a, b; Yang et al. 2020; Ye et al. 2010). *P. notoginseng* saponins (PNS) are the main active components of *P. notoginseng*. Among them, ginsenosides Rg_1_, Rb_1_, Rd, Re and notoginsenoside R_1_ are the major saponins, accounting for over 80% of the PNS. Saponins have pharmacological effects, such as anti-inflammatory, antioxidant, inhibition of platelet aggregation, regulation of blood glucose and blood pressure, inhibition of neuronal apoptosis, protection of neurons, etc. (Duan et al. 2018; Lin et al. 2015; Wei et al. 2023; Xiong et al. 2019). However, the major ginsenosides in PNS (including Rg_1_, Rb_1_, Rd, Re, R_1_) are difficult to be absorbed by the human body due to their high molecular weight, low membrane permeability, and low bioavailability (Cui et al. 2016; Upadhyaya et al. 2016). After oral administration, they need to be converted into minor ginsenosides by human gut microbiota and gastric juice before they can be readily absorbed into the bloodstream and exert their effects. In addition, pharmacological studies showed that rare saponins have better biological activity, but their content in *P. notoginseng* is very low (Park et al. 2010; Wei et al. 2011; Wu et al. 2012). Thus, a lot of studies have been focused on the conversion of major ginsenosides to rare ginsenosides (Li et al. 2022). Biotransformation is the most promising method to produce rare ginsenosides, which has the advantages of strong specificity, high yield, low cost and environmental friendliness (Zhang et al. 2023a, b; Li et al. 2022a, b). Studies showed that the genus *Aspergillus* has the ability of transformation saponins to rare ginsenosides. Such as *Aspergillus tubingensis* can convert ginsenoside Rb_1_, Rb_2_, Rc, and Rd to CK. (Song et al. 2023); *Aspergillus Niger* XD101 can convert ginsenoside Rb_1_ to CK (Jiang et al. 2021). However, there have no reports on the separation, purification, and structural identification of PNS transformation products by the genus of *Aspergillus.* This study aimed to transform major ginsenosides into rare ginsenosides from PNS using fungus *Aspergillus fumigatus*, and to speculate their transformation pathways. This study reported for the first time the microbial conversion of PNS using *A. fumigatus,* and 14 rare ginsenosides were isolated from the converted products. This paper provides a new microbial conversion strain source for the large-scale preparation of rare ginsenosides, and also provides a theoretical basis for improving the medicinal value of *P. notoginseng*.

## Materials and methods

### Strains

The strain of *A. fumigatus* was isolated from fresh *P. notoginseng* root soil in our previous research, and was conserved in Potato Dextrose Agar (PDA) medium. A voucher specimen (No. Yang20210907) was deposited at the Faculty of Life Science and Technology, Kunming University of Science and Technology.

### Sample, chemical, and reagents

Reference standards, including ginsenosides Rg_1_, Re, Rb_1_, Rd, 20 (*S/R*)-Rg_2_, 20 (*R*)-Rh_1_, Rg_6_, Rk_3_, Rh_4_, 20 (*S/R*)-Rg_3_, Rk_1_, Rg_5_, CK, 20 (*S/R*)-Rh_2_, and notoginsenosides R_1_, 20 (*S/R*)-R_2_ were purchased from the Sichuan Victory Biological Technology Co., Ltd. (Sichuan, China). PNS was supported by professor of Xiuming Cui, Kunming University of Science and Technology. The solvents methanol and acetonitrile for HPLC were purchased from Sigma-Aldrich Co. (St. Louis, MO, USA). The Welchrom C_18_ Column (4.6 × 250 mm, 5 µm) was purchased from Yuexu Technology Co, Ltd. (Sichuan China). The Agilent 1260 High Performance liquid chromatograph was purchased from Agilent (Grand Island, NY, USA). The GF_254_ Silica gel plate was purchased from Qingdao Marine Chemical Plant Co., Ltd. (Shandong, China). The standard strains, *Staphylococcus aureus* (CMCC(B)26003) and *Candida albicans* (BNCC109047), were purchased from Engineering Research Center of Industrial Microbiology (Henan, China). Ciprofloxacin was purchased from Beijing Solarbio Science & Technology Co., LTD (Beijing, China).

### Morphological observation of *A. fumigatus*

*A. fumigatus* was inoculated on PDA medium and cultured in 25 ℃ incubator for 3 days. Colony characteristics and morphological characteristics (under optical microscope) were observed.

### Medium

PDA medium: potato extract powder 5 g/L, glucose 15 g/L, and agar 25 g/L. PDB medium: potato extract powder 5 g/L and glucose 15 g/L.

### Microbial transformation of PNS by *A. fumigatus*

We transferred well-developed fungal hyphae from the surface of the agar slant to three 500 mL Erlenmeyer flasks containing 300 mL PDB medium. The cultures were grown for 3 d on a rotating shaker at 25 °C with shaking at 150 rpm to produce seed liquid. Then, the seed liquids were transferred to 500 mL reagent bottles that contained 300 mL of medium for expand fermentation. The cultures were then incubated using the same conditions as before. After 4 d, PNS (transformation substrate) were added to the cultures at the concentration of 5 mg/mL. The cultures were incubated for additional 46 days at 25 °C with shaking at 150 rpm. Finally, A total of 172.5 g PNS was transformed and a total of 34.5 L fermentation broth were prepared. The mycelia were separated by filtration and the filtrate was extracted five times with n-butanol. The organic layer was concentrated under reduced pressure to afford a residue (161.5 g).

### Chromatographic conditions

A total of 161.5 g residue was obtained from the fermentation solution. D101 macroporous resin column chromatography (H_2_O and EtOH as mobile phase, 0%→20%→40%→60%→80%→100% EtOH), silica gel column chromatography (CH_2_Cl_2_ and MeOH as mobile phase, CH_2_Cl_2_:MeOH = 20:1→15:1→10:1→8:1→5:1→3:1→1:1→0:1), Rp-C_18_ reversed phase column chromatography (MeOH and H_2_O as mobile phase, 20%→40%→60%→80%→100% MeOH), semi-preparative HPLC (MeOH and H_2_O as mobile phase, 50%–100% MeOH, the wavelength is 203 nm), Sephadex LH-20 gel column chromatography (CH_2_Cl_2_:MeOH = 1:1) and other purification methods. The structures of the compounds were elucidated on the basis of their ^1^H-NMR, ^13^C-NMR and ESIMS spectroscopic data.

### Analysis the conversion rate and products yield by HPLC

HPLC was performed using an Agilent 1260 system (Grand Island, NY, USA). A reverse phase column (4.6 × 250 mm, 5 µm; Yuexu Technology Co., Ltd. Sichuan China) at 30 ℃ was used. To determine the conversion rate of five main ginsenosides of PNS and the yield of conversion products, the conversion substrate (PNS), conversion products (extract), and saponin standards were dissolved in methanol prepared for analyzed by HPLC. H_2_O and CH_3_CN were used as the mobile phases A and B, respectively. The gradient elution was programmed as follows: 0–30 min, 20% (B); 30–60 min, 20–37% (B); 60–65 min, 37–38% (B); 65–70 min, 38–45% (B); 70–75 min, 45–50% (B); 75–90 min, 50–56% (B); 90–93 min, 56–62% (B); 93–103 min, 62–75% (B). The flow rate and detection wavelength were set as 1 mL/min and 203 nm respectively. The injection volume was 30 μL.

### Antimicrobial activity

Transformation products (**1**–**14**) were evaluated for antimicrobial activity against 2 human pathogenic microbia. The tested microbia were: *Staphylococcus aureus and Candida albicans.* The activity of compounds **1**–**14** against human pathogenic microbia was tested by double dilution method. Compounds **1**–**14** and positive control ciprofloxacin were dissolved with DMSO and the concentration was 1 mg/mL. Pathogenic microbia were incubated in LB medium at 37 ℃ and 160 rpm for 8–12 h, microbia solution and compounds **1**–**14** were added to 96-well plates, incubated at 37 ℃, and the 96-well plate was observed to be clear and transparent without microbia growth after 12 h. The experiment was repeated three times. LB broth medium component: 20 g LB Broth dissolved in 1000 mL distilled water.

## Results and discussion

### Morphological observation of *A. fumigatus*

**Fig. 1:**
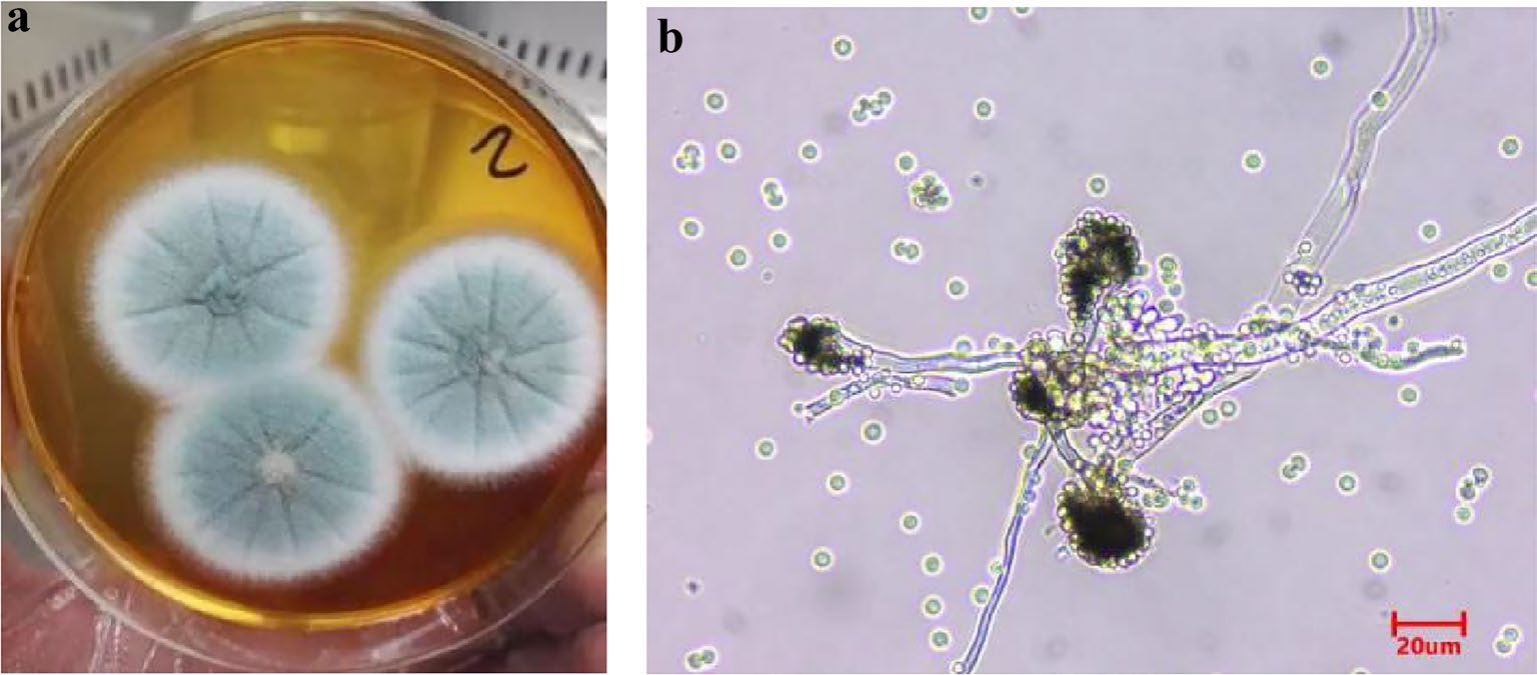
Morphology of *A*. *fumigatus*. **a** Colony morphology diagram; **b** Spore map of *A*. *fumigatus*

*A. fumigatum* was inoculated on PDA and cultured in 25 ℃ incubator for 3 days. The following colony characteristics were observed: *A. fumigatus* is fluffy or flocculent, dark green (Fig. 1a). The conidial head is columnar, green when young, dark green when mature; The surface of the conidial stem is smooth, and many spores are scattered around it (Fig. 1b).

### Separation of transformation products

The 161.5 g residue was eluted by D101 macroporous resin column chromatography with a gradient elution of an ethanol–water solvent system to obtain four fractions (Frs A ~ D). Fr D was further obtained by silica gel column chromatography (CH_2_Cl_2_/MeOH = 20:1→15:1→10:1→5:1→1:1) to obtain four components Frs D_1_-D_4_. Two components Fr D_1-1_-Fr D_1-2_ were obtained by Rp-C_18_ reversed phase column chromatography (MeOH/H_2_O = 3:7→1:1→7:3→1:0) from Fr D_1_. The compounds **1** (7.0 mg) and **3** (19.3 mg) were obtained from Fr D_1a_ by Sephadex LH-20 gel column chromatography (CH_2_Cl_2_/MeOH = 1:1). The compounds **5** (t_R_ = 13.2 min, 9 mg), **7** (t_R_ = 17.5 min, 50.5 mg) and **11** (t_R_ = 23.5 min, 8 mg) were obtained from Fr D_1b_ by semi-preparative HPLC (MeOH/H_2_O: 70–90%, 30 min, 2 mL/min). The compounds **12** (t_R_ = 6.8 min, 3 mg) and **14** (t_R_ = 12.4 min, 6 mg) was obtained from Fr D_2_ by semi-preparative HPLC (MeOH/H_2_O: 65–80%, 20 min, 2 mL/min). Fr D_3_ was subjected to Sephadex LH-20 gel column chromatography (CH_2_Cl_2_/MeOH = 1:1) to obtain two subfractions Fr D_3a_ and Fr D_3b_. The compounds **2** (9.5 mg), **6** (10 mg) and **13** (2 mg) were obtained by silica gel column chromatography (CH_2_Cl_2_/MeOH = 7:1→4:1→2:1) from Fr D_3a_. The compounds **4** (t_R_ = 9.4 min, 6 mg) and **8** (t_R_ = 15.4 min, 6 mg) were obtained from Fr D_3b_ by semi-preparative HPLC (MeOH/H_2_O: 55%–70%, 25 min, 2 mL/min). The compounds **9** (t_R_ = 17.5 min, 2 mg) and **10** (t_R_ = 23.7 min, 13 mg) were obtained from Fr D_4_ by semi-preparative HPLC (MeOH/H_2_O: 50%-70%, 35 min, 2 mL/min). semi-preparative HPLC conditions are as follows: H_2_O and MeOH were used as the mobile phases A and B, respectively. The wavelength is 203 nm. The detailed flowchart as shown in Fig. S17.

### Structural characterization of products

**Fig. 2:**
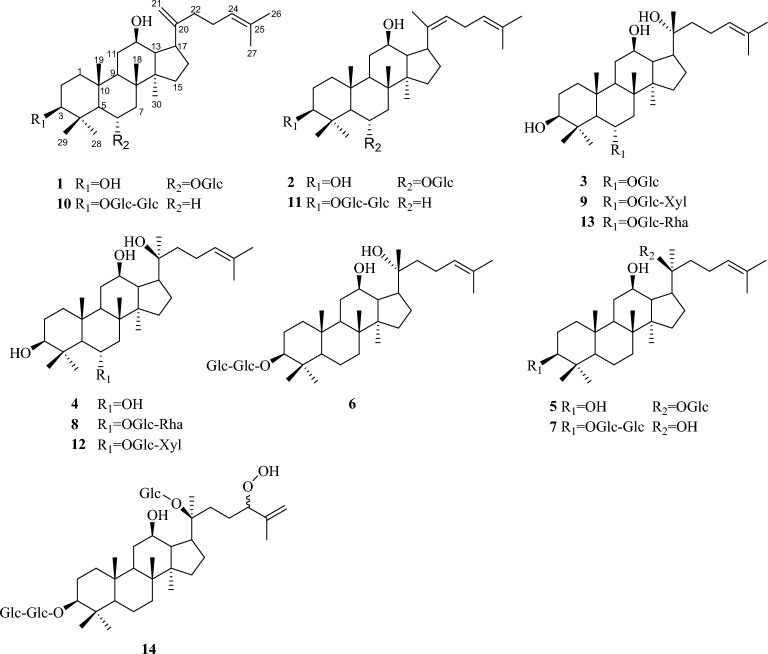
Structures of minor ginsenosides from microbial transformation of PNS by *Aspergillus fumigatus*

The structures of products were identified on the basis of their spectroscopic data. Data of ^1^H and ^13^C NMR spectra of products **1**–**14** were in agreement with the reported literatures’ data. Compounds **1**–**14** were identified as ginsenoside Rk_3_ (**1**) (Park et al. 2002), Rh_4_ (**2**) (Park et al. 2002), 20 (*R*)-Rh_1_ (**3**) (Teng et al. 2002), 20 (*S*)-Protopanaxatriol (**4**) (Usami et al. 2008), C-K (**5**) (Zhou et al. 2009), 20 (*R*)-Rg_3_ (**6**) (Teng et al. 2004), 20 (*S*)-Rg_3_ (7) (Teng et al. 2004), 20 (*S*)-Rg_2_ (**8**) (Wang et al. 2007), notoginsenoside 20 (*R*)-R_2_ (**9**) (Chen et al. 2007), ginsenoside Rk_1_ (**10**) (Park et al. 2002), Rg_5_ (**11**) (Kim et al. 1996), notoginsenoside 20 (*S*)-R_2_ (**12**) (Teng et al. 2002), ginsenoside 20 (*R*)-Rg_2_ (**13**) (Yang et al. 2000), and 20 (*S*)-I (**14**) (Yoshikawa et al. 1997). The structures of isolated rare ginsenosides (**1**–**14**) as shown in Fig. 2. The detailed ^1^H-NMR and ^13^C-NMR data of compounds **1**–**14** were shown in Table S1-S7.

### Conversion rate of four products of PNS

The conversion rate of substrates and the yield of products was defined as follows:

Conversion rate (%) = $$\frac{m-{m}_{1}}{m}$$  × 100%

Productivity (%) = $$\frac{{m}_{2}}{m}$$  × 100%

m: the mass of substrate; m_1_: the mass of remaining substrate; m_2_: the mass of products.

The major ginsenosides of PNS was notoginsenoside R_1_, ginsenosides Rg_1_, Re, and Rb_1_, the conversion rate of them were 32.52, 19.35, 24.74, and 100%, respectively. The conversion products and productivity were shown in Table 1.

**Table 1:** The conversion products and their productivity

Substrates	Transformation rate (%)	Products	Productivity (%)
Rb_1_	100	20(*S*)-Rg_3_	17.90
		20(*R*)-Rg_3_	22.29
		Rg_5_	6.30
		Rk_1_	3.25
		CK	0.19
		20(*S*)-I	5.60
Rg_1_	19.35	Rk_3_	3.61
		Rh_4_	6.42
R_1_	32.52	**—**	**—**
Re	24.74	**—**	**—**

### Propose possible biosynthetic pathways *of major* ginsenosides Rg_1_, Re, Rb_1_ and notoginsenoside R_1_ of PNS

The transformation pathway of ginsenoside Rb_1_ is proposed in Fig. S1A. Rb_1_ obtained Rd after hydrolyzing the lateral glucose of C-20, so the two monomers share the same conversion pathway. The ginsenoside Rb_1_ contains four glucopyranosyl moieties at the C-3 and C-20 position of aglycone. According to the isolated ginsenosides 20 (*R/S*)-Rg_3_, Rg_5_ and Rk_1_, C-K, 20 (*S*)-I, the conversion pathway of Rb_1_ can be predicted. The first pathway is the *A. fumigatus* attacked the outer *β*-(1 → 6)-glucosidic bond linkages to C-20 position of aglycone to produce Rd from Rb_1_, and was then followed by the hydrolysis of the outer *β*-(1 → 6)-glucosidic bond to C-3 position to produce F_2_, later followed by the hydrolysis of the inner *β*-(1 → 6)-glucosidic bond to C-3 position to produce CK or by heating and oxidizing the air to get 20 (*S*)-I. Another pathway was followed by the hydrolysis of the inner *β*-(1 → 6)-glucosidic bond to the C-20 position to produce 20 (*R/S*)-Rg_3_ from Rd, then through dehydration reaction at the C-20 position to form a double bond with C-21 to get Rk_1_, or to form a double bond with C-22 to get Rg_5_.

The transformation pathway of ginsenoside Rg_1_ is proposed in Fig. S1B. Using the same method as above, we proposed the pathway of ginsenoside Rg_1_ as follows: Rg_1_→20 (*R*)-Rh_1_→Rh_4_; Rg_1_→20 (*R*)-Rh_1_→20 (*S*)-protopanaxatriol; Rg_1_→20 (*R*)-Rh_1_→Rk_3_, respectively (Fig. S1B). We also proposed the pathway of ginsenoside Re as follow: Re→20 (*R/S*)-Rg_2_→20 (*R*)-Rh_1_→20 (*S*)-protopanaxatriol (Fig. S1C). Lastly, we proposed the pathway of notoginsenoside R_1_ as follow: R_1_→20 (*R/S*)-R_2_→20 (*R*)-Rh_1_→20 (*S*)-protopanaxatriol (Fig. S1D).

### Antimicrobial activity

The antimicrobial activities of compounds **1**–**14** against 2 pathogenic microbial were tested. As shown in Table 2, Compounds **5** and **7** have moderate antimicrobial activity against *Staphylococcus aureus* and *Candida albicans*, with MIC values of 6.25, 1.25 μg/mL and 1.25, 25 μg/mL, respectively. Additionally, compounds **10** and **11** also had certain antimicrobial activity against *Staphylococcus aureus* and *Candida albicans*. The antimicrobial activity of compounds **1**–**14** as shown in Table 2

**Table 2:** Antimicrobial activity of compounds **1**–**14** (MIC, *μ*g/mL)

Compounds	pathogenic microbia	
	*Staphylococcus aureus*	*Candida albicans*
**1**	> 100	> 100
**2**	> 100	> 100
**3**	> 100	> 100
**4**	> 100	> 100
**5**	6.25	1.25
**6**	> 100	> 100
**7**	1.25	25
**8**	> 100	> 100
**9**	> 100	> 100
**10**	25	25
**11**	50	50
**12**	> 100	> 100
**13**	> 100	> 100
**14**	> 100	> 100
ciprofloxacin	1.56	0.78

## Conclusion

Rare ginsenosides is a group of dammarane triterpenoids that exist in low natural abundance, which can be produced by physicochemical processing or metabolic transformation of major ginsenosides. Due to their small polarity and molecular weight, they exhibited potent biological activity comparing to the primary ginsenosides. The fungus *A. fumigatus* has the ability of transform PNS to rare ginsenosides. We isolated 14 rare ginsenosides from the transformation products. The structure analysis of 14 rare ginsenosides showed that they were the metabolites of ginsenosides Rb_1_, Rg_1_, Rd, Re and notoginsenoside R_1_ (they are the major ginsenosides of PNS), respectively. Based on the structure of the transformation products, we speculate on the possible biological transformation pathways of saponins (Fig. S1). The conversion rates of four ginsenosides were calculated by HPLC analysis, and it was found that the conversion rates of ginsenosides Rb_1_, R_1_, Rg_1_, Re were 100, 32.52, 19.35, 24.74%, respectively. The yield of ginsenoside 20 (*R*)-Rg_3_, which has good anti-tumor effect, can reach 22.29%. The yield of other ginsenosides 20 (*S*)-Rg_3_, Rh_4_, 20 (*S*)-I, Rg_5_, Rk_3,_ Rk_1_ and C-K could reach 17.90, 6.42, 5.60, 6.30, 3.61, 3.25 and 0.19%, respectively. We found that the products of PNS converted by *A.fumigatus* were rich and varied, and the conversion rate of ginsenoside Rb_1_ could reach 100%, which completely transformed components in PNS.

Our study found that the process of PNS transformed by *A.fumigatus*, involved a variety of reactions, including deglycosylation, dehydration, and oxygenation. So, we can obtain multiple products through transformation from PNS by this fungus. Compared with other studies (Song et al. 2023 and Jiang et al. 2021), our transformation products are more abundant, we can not only obtain Rg_3_ and CK as other literatures, but also can obtain many other rare ginsenosides (Rh_4_, 20 (*S*)-I, Rg_5_, Rk_3,_ Rk_1_, et al.). So, this study provided an active fungus to prepare diversity rare ginsenosides. Besides this, we deduced the transformation pathway of saponins, which can provide theoretical basis for the acquisition of target rare ginsenosides.

Through the transformation of PNS by *A. fumigatus*, this study can provide a method for obtaining the rare saponins, lay a foundation for the efficient utilization *P. notoginseng*, improve the pharmacological activity and economic value of saponins in *P. notoginseng*, and provide a basis and theoretical support for large-scale industrial preparation of rare saponins.

### Supplementary Information

Additional file1 Fig. S1. Proposed possible biosynthetic pathways of major ginsenosides Rg_1_, Re, Rb_1_ and notoginsenoside R_1_ of PNS by *A. fumigatus*. Fig. S2. HPLC analysis of transformation products of PNS by *A. fumigatus*. Table S1~S7 ^1^H and ^13^C NMR spectral data of compounds **1**-**14**. Figs. S3~S16. ^1^H NMR and ^13^C NMR (C_5_D_5_N) of compounds **1**-**14**. Fig. S17. Separation flow diagram of compounds **1**-**14**. Fig. S18. TLC analysis of transformation products at different times during the conversion process.

## Data Availability

The data and the materials are all available in this article and its supporting information document, which will be given access on the journal’s website.

## Abbreviations

PNS: *Panax notoginseng* Saponins, mainly including notoginsenoside R_1_, ginsenosides Rg_1_, Re, Rb_1_, Rd
TLC: Thin layer chromatography
HPLC: High performance liquid chromatography
PDA: Potato Dextrose Agar
PDB: Potato Dextrose Broth
MIC: Minimum inhibitory concentration
