# Supplementary material for: Production of rare ginsenosides by biotransformation of Panax notoginseng saponins using Aspergillus fumigatus
Source: Bioresour Bioprocess. 2024 Aug 9;11(1):81. doi: 10.1186/s40643-024-00794-0 (PMC11319572; doi:10.1186/s40643-024-00794-0)
Supplement: Supplementary file 1 — Additional file1 Fig. S1. Proposed possible biosynthetic pathways of major ginsenosides Rg1, Re, Rb1 and notoginsenoside R1 of PNS by A. fumigatus. Fig. S2. HPLC analysis of transformation products of PNS by A. fumigatus. Table S1~S7 1H and 13C NMR spectral data of compounds 1-14. Figs. S3~S16. 1H NMR and 13C NMR (C5D5N) of compounds 1-14. Fig. S17. Separation flow diagram of compounds 1-14. Fig. S18. TLC analysis of transformation products at different times during the conversion process. [file 40643_2024_794_MOESM1_ESM.docx]

**Supporting Information**

**Production of rare ginsenosides by biotransformation of *Panax notoginseng* saponins using** ***Aspergillus fumigatus***

Lian Yang^1^, Dongmei Lin^1^, Feixing Li^1^, Xiuming Cui^1^, Dengji Lou^2*^, Xiaoyan Yang^1^*^[[1]](#footnote-1)^

**Abstract**

*Panax notoginseng* saponins (PNS) are the main active components of *Panax notoginseng*. But after oral administration, they need to be converted into rare ginsenosides by human gut microbiota and gastric juice before they can be readily absorbed into the bloodstream and exert their effects. The sources of rare ginsenosides are extremely limited in *P. notoginseng* and other medical plants, which hinders their application in functional foods and drugs. Therefore, the production of rare ginsenosides by the transformation of PNS using *Aspergillus fumigatus* was studied in this research. During 50 days at 25 ℃ and 150 rpm, *A. fumigatus* transformed PNS to 14 products (**1**-**14**). They were isolated by varied chromatographic methods, such as silica gel column chromatography, Rp-C_18_ reversed phase column chromatography, semi-preparative HPLC, Sephadex LH-20 gel column chromatography, and elucidated on the basis of their ^1^H-NMR, ^13^C-NMR and ESIMS spectroscopic data. Then, the transformed products (**1**-**14**) were isolated and identified as Rk_3_, Rh_4_, 20 (*R*)-Rh_1_, 20 (*S*)-Protopanaxatriol, C-K, 20 (*R*)-Rg_3_, 20 (*S*)-Rg_3_, 20 (*S*)-Rg_2_, 20 (*R*)-R_2_, Rk_1_, Rg_5_, 20 (*S*)-R_2_, 20 (*R*)-Rg_2_, and 20 (*S*)-I, respectively. In addition, all transformed products (**1**-**14**) were tested for their antimicrobial activity. Among them, compounds **5** (C-K) and **7** [20 (*S*)-Rg_3_] showed moderate antimicrobial activities against *Staphylococcus aureus* and *Candida albicans* with MIC values of 6.25, 1.25 μg/mL and 1.25, 25 μg/mL, respectively. This study lays the foundation for production of rare ginsenosides.

**Keywords** *Panax notoginseng* saponins, biotransformation, rare ginsenosides, *Aspergillus fumigatus*, antimicrobial activity

**Content**

[**Figure S1**. Proposed possible biosynthetic pathways of major ginsenosides Rg_1_, Re, Rb_1_ and notoginsenoside R_1_ of PNF by *A*. *fumigatus* 3](#_Toc93566642)

[**Figure S2**. HPLC analysis of transformation products of PNF by *A*. *fumigatus* 4](#_Toc93566642)

[**Figure S3**. ^1^H-NMR and ^13^C-NMR spectra of compound **1** 5](#_Toc93566642)

[**Figure S4**. ^1^H-NMR and ^13^C-NMR spectra of compound **2** 6](#_Toc93566642)

[**Figure S5**. ^1^H-NMR and ^13^C-NMR spectra of compound **3** 7](#_Toc93566642)

[**Figure S6**. ^1^H-NMR and ^13^C-NMR spectra of compound **4** 8](#_Toc93566642)

[**Figure S7**. ^1^H-NMR and ^13^C-NMR spectra of compound **5** 9](#_Toc93566642)

[**Figure S8**. ^1^H-NMR and ^13^C-NMR spectra of compound **6** 10](#_Toc93566642)

[**Figure S9**. ^1^H-NMR and ^13^C-NMR spectra of compound **7** 11](#_Toc93566642)

[**Figure S10**. ^1^H-NMR and ^13^C-NMR spectra of compound **8** 12](#_Toc93566642)

[**Figure S11**. ^1^H-NMR and ^13^C-NMR spectra of compound **9**](#_Toc93566642) 13

[**Figure S12**. ^1^H-NMR and ^13^C-NMR spectra of compound **10** 14](#_Toc93566642)

[**Figure S13**. ^1^H-NMR and ^13^C-NMR spectra of compound **11** 15](#_Toc93566642)

[**Figure S14**. ^1^H-NMR and ^13^C-NMR spectra of compound **12** 16](#_Toc93566642)

[**Figure S15**. ^1^H-NMR and ^13^C-NMR spectra of compound **13** 17](#_Toc93566642)

[**Figure S16**. ^1^H-NMR and ^13^C-NMR spectra of compound **14** 18](#_Toc93566642)

[**Figure S17**. Separation flow diagram of compounds **1**-**14** 19](#_Toc93566642)

[**Figure S18**. TLC analysis of transformation products at different times during the conversion process 19](#_Toc93566642)

[**Table S1**. ^1^H and ^13^C NMR spectral data of **1**-**2** 20](#_Toc93566642)

[**Table S2**. ^1^H and ^13^C NMR spectral data of **3**-**4** 21](#_Toc93566642)

[**Table S3**. ^1^H and ^13^C NMR spectral data of **5**-**6** 22](#_Toc93566642)

[**Table S4**. ^1^H and ^13^C NMR spectral data of **7**-**8** 23](#_Toc93566642)

[**Table S5**. ^1^H and ^13^C NMR spectral data of **9**-**10** 24](#_Toc93566642)

[**Table S6**. ^1^H and ^13^C NMR spectral data of **11**-**12** 25](#_Toc93566642)

[**Table S7**. ^1^H and ^13^C NMR spectral data of **13**-**14** 26](#_Toc93566642)

**Fig. S1** Proposed possible biosynthetic pathways of major ginsenosides Rg_1_, Re, Rb_1_ and notoginsenoside R_1_ of PNF by *A*. *fumigatus*. The solid line represents the determined path, and the dashed line represents the speculated path.

**Fig. S2** HPLC analysis of transformation products of PNF by *A*. *fumigatus*. a, Rare ginsenosides mixed; b, Sample of PNS after transformation; c: PNS. 1-5, R_1_, Rg_1_, Re, Rb_1_, Rd. 6, 20 (*S*)-I; 7+8, 20 (*S*)-R_2_+20 (*S*)-Rg_2_; 9, 20 (*R*)-Rg_2_+20 (*R*)-R_2_; 10, 20 (*R*)-Rh_1_; 11, Rk_3_; 12, Rh_4_; 13, 20 (*R*)-Rg_3_; 14, 20 (*S*)-Rg_3_; 15, 20 (*S*)-Protopanaxatriol; 16, Rk_1_; 17, Rg_5_; 18, CK.


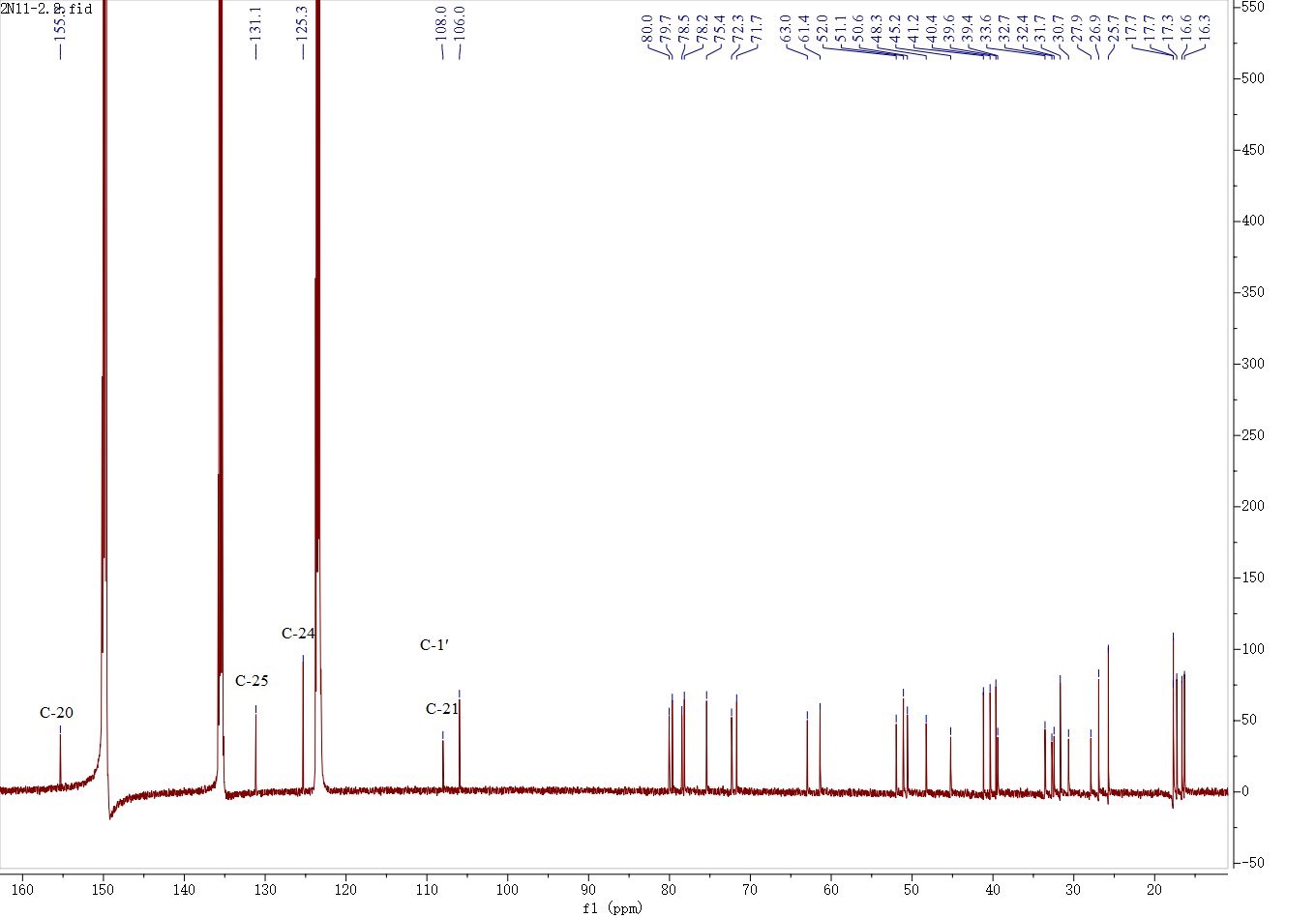

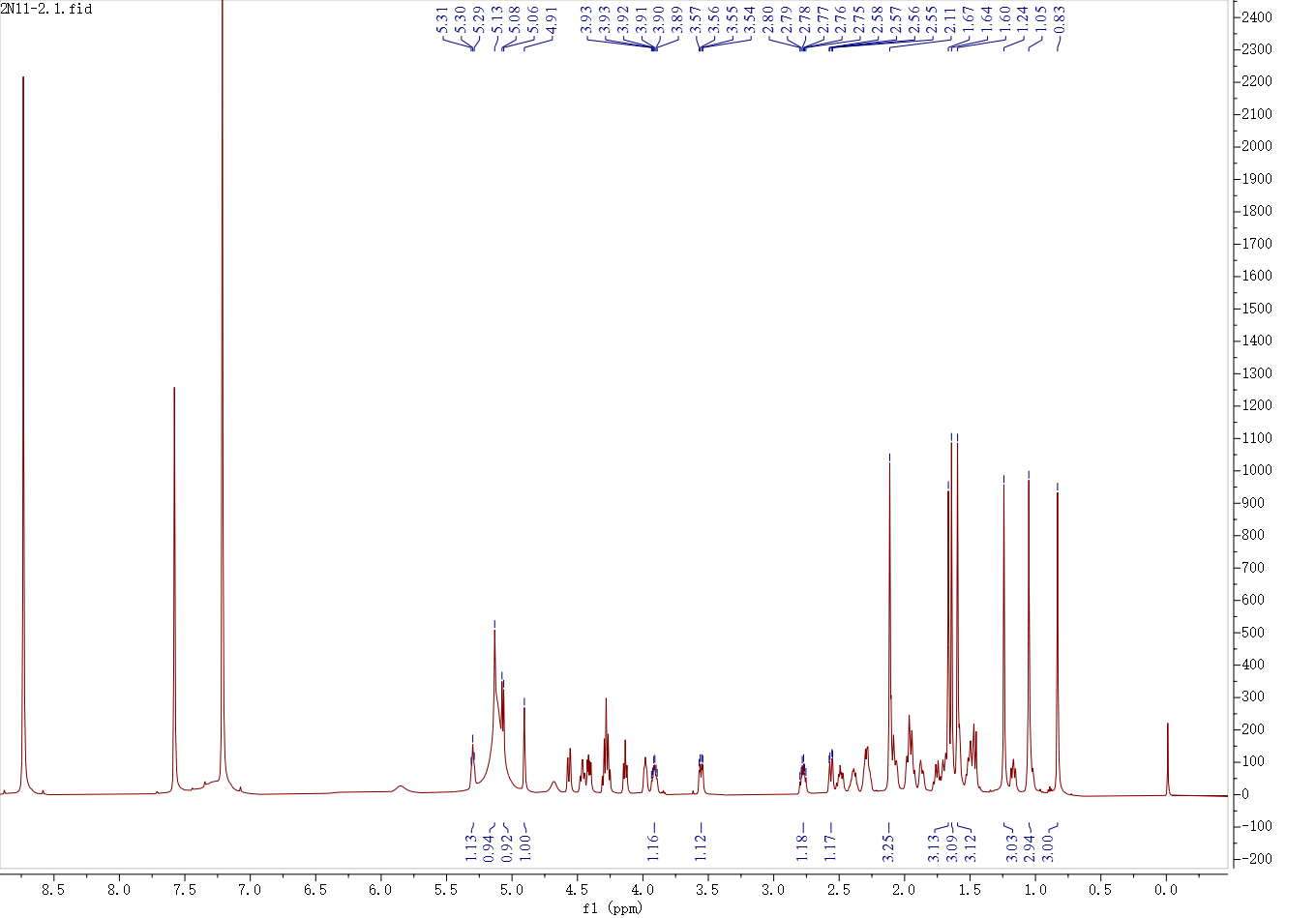


**Fig. S3** ^1^H-NMR and ^13^C-NMR spectra of compound **1**

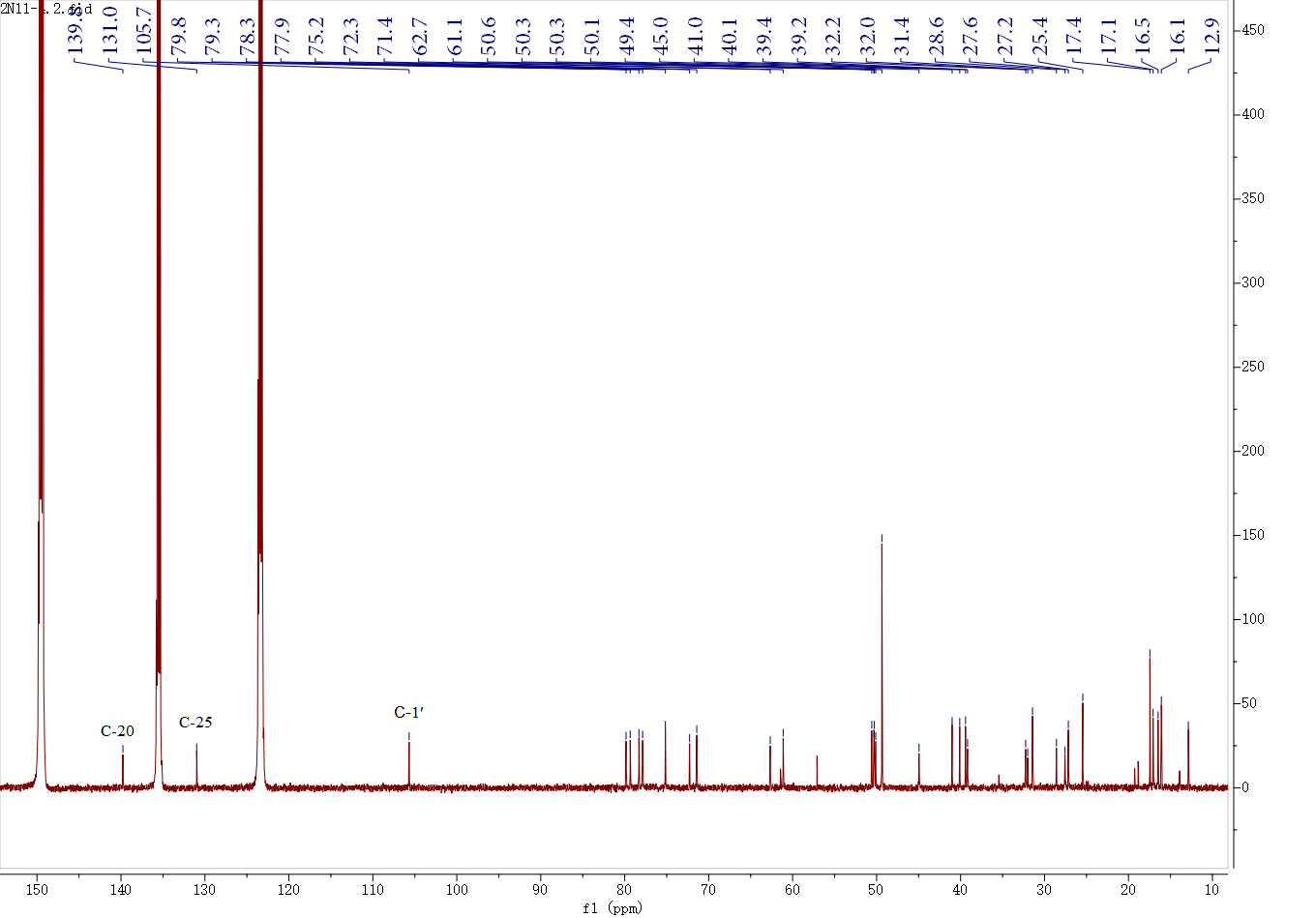
**Fig. S4** ^1^H-NMR and ^13^C-NMR spectra of compound **2**


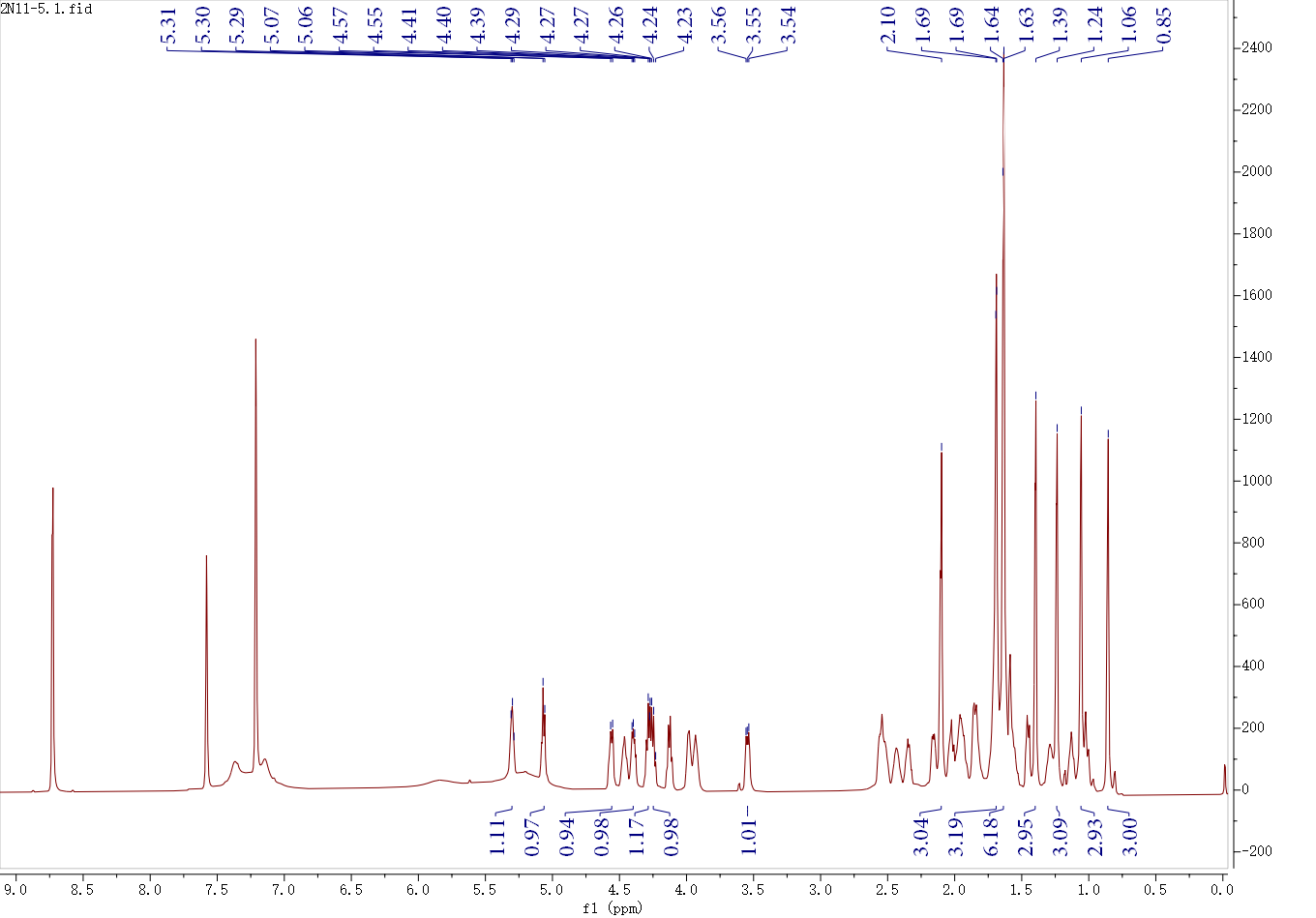


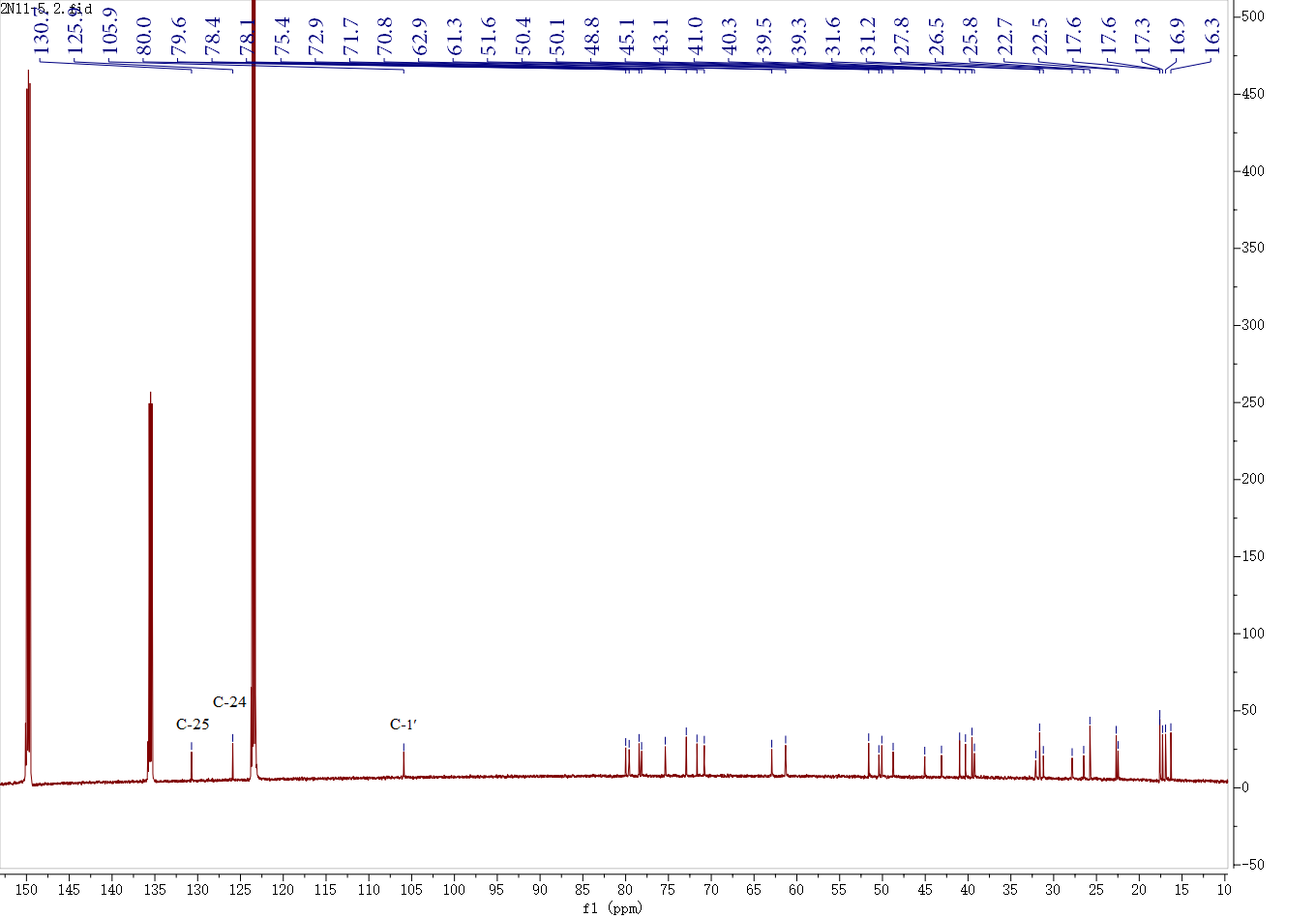


**Fig. S5** ^1^H-NMR and ^13^C-NMR spectra of compound **3**


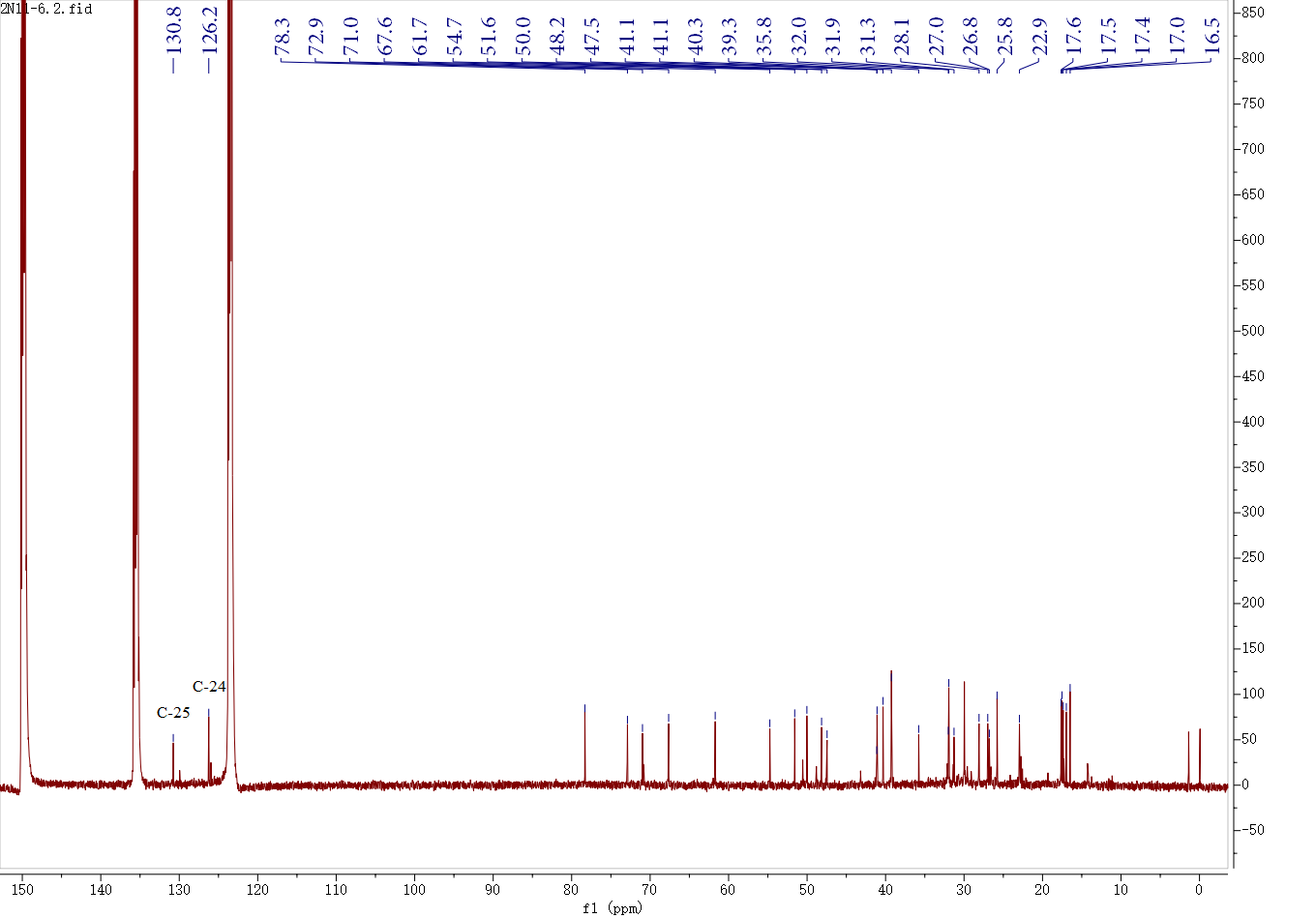

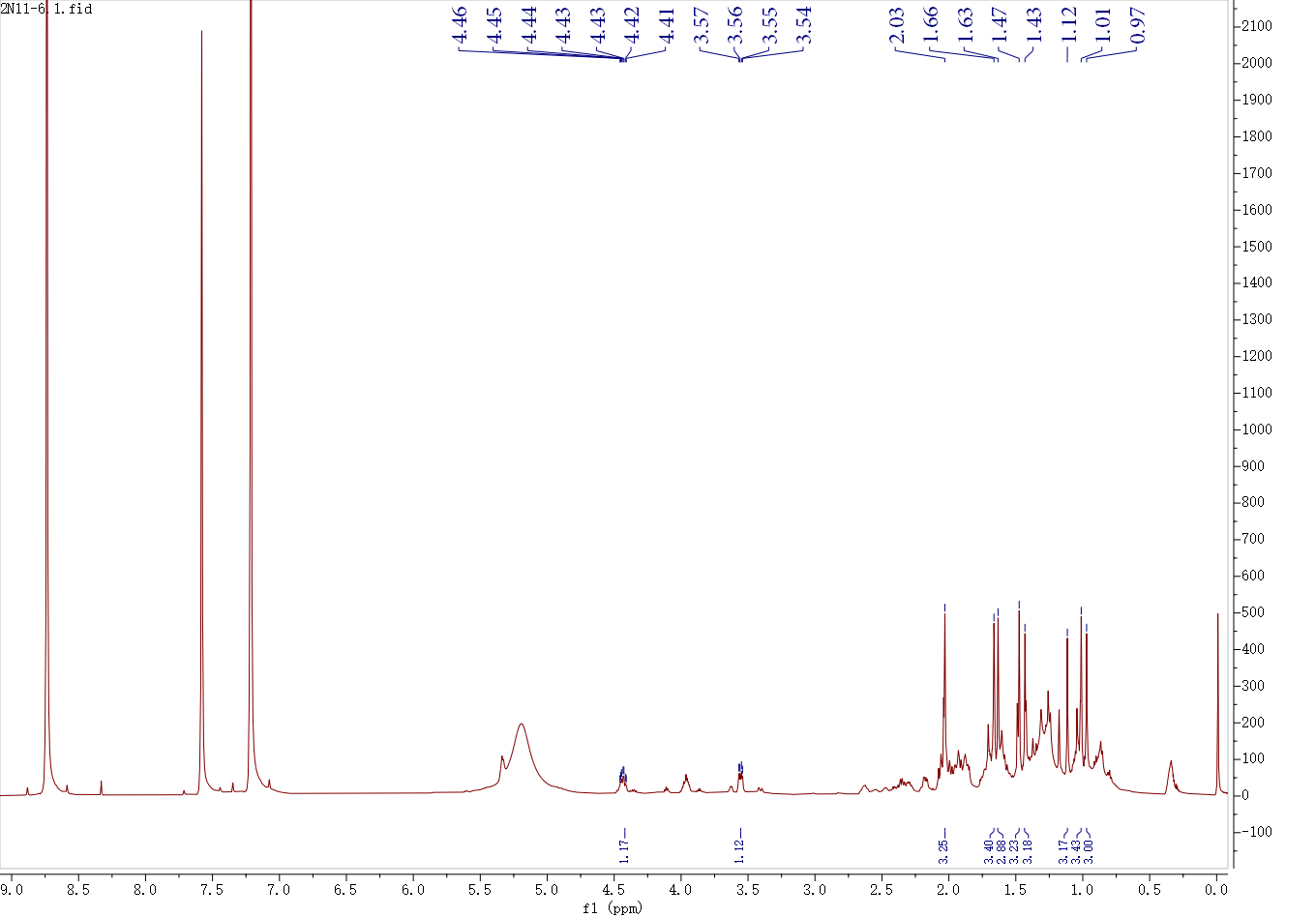


**Fig. S6** ^1^H-NMR and ^13^C-NMR spectra of compound **4**


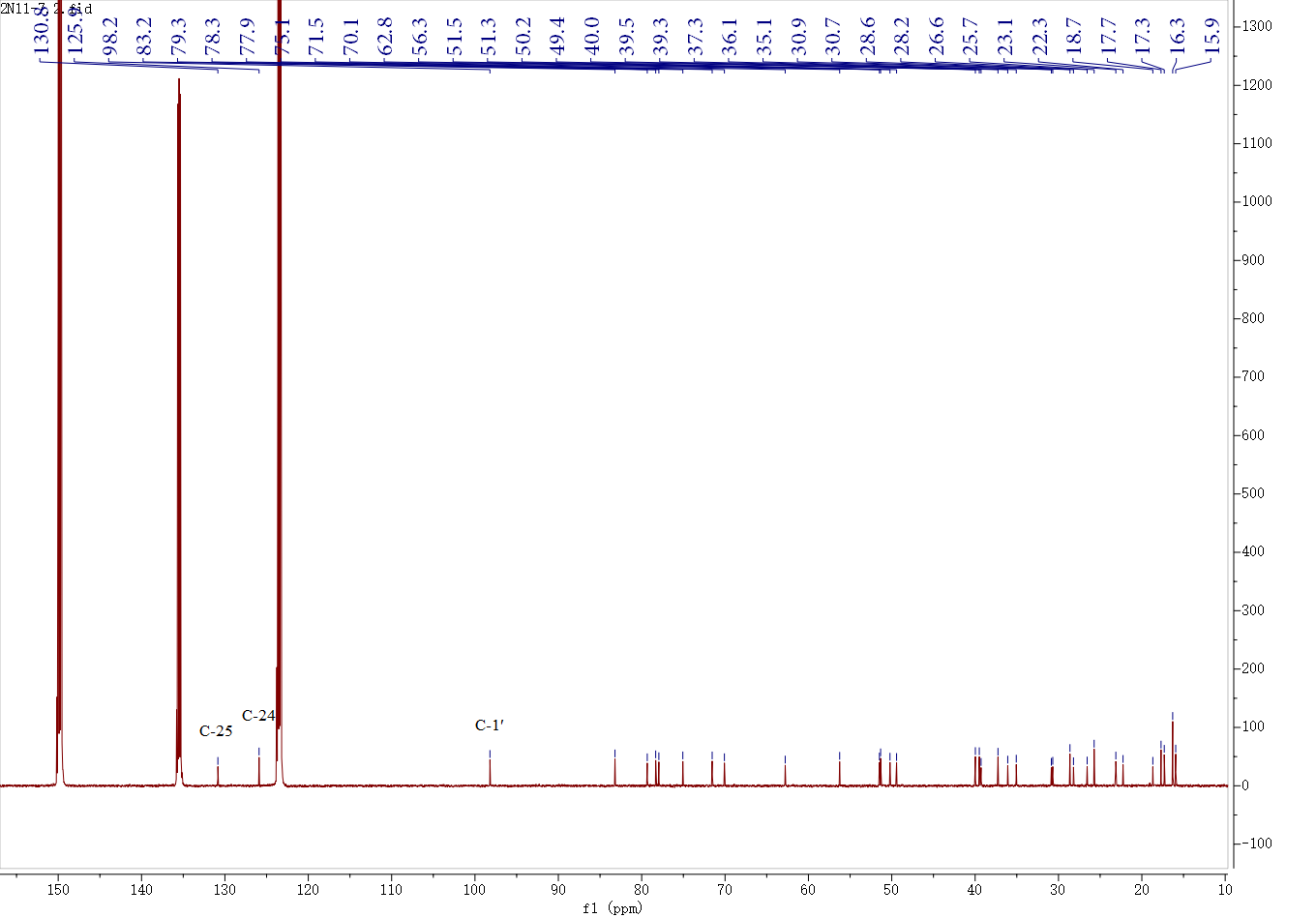

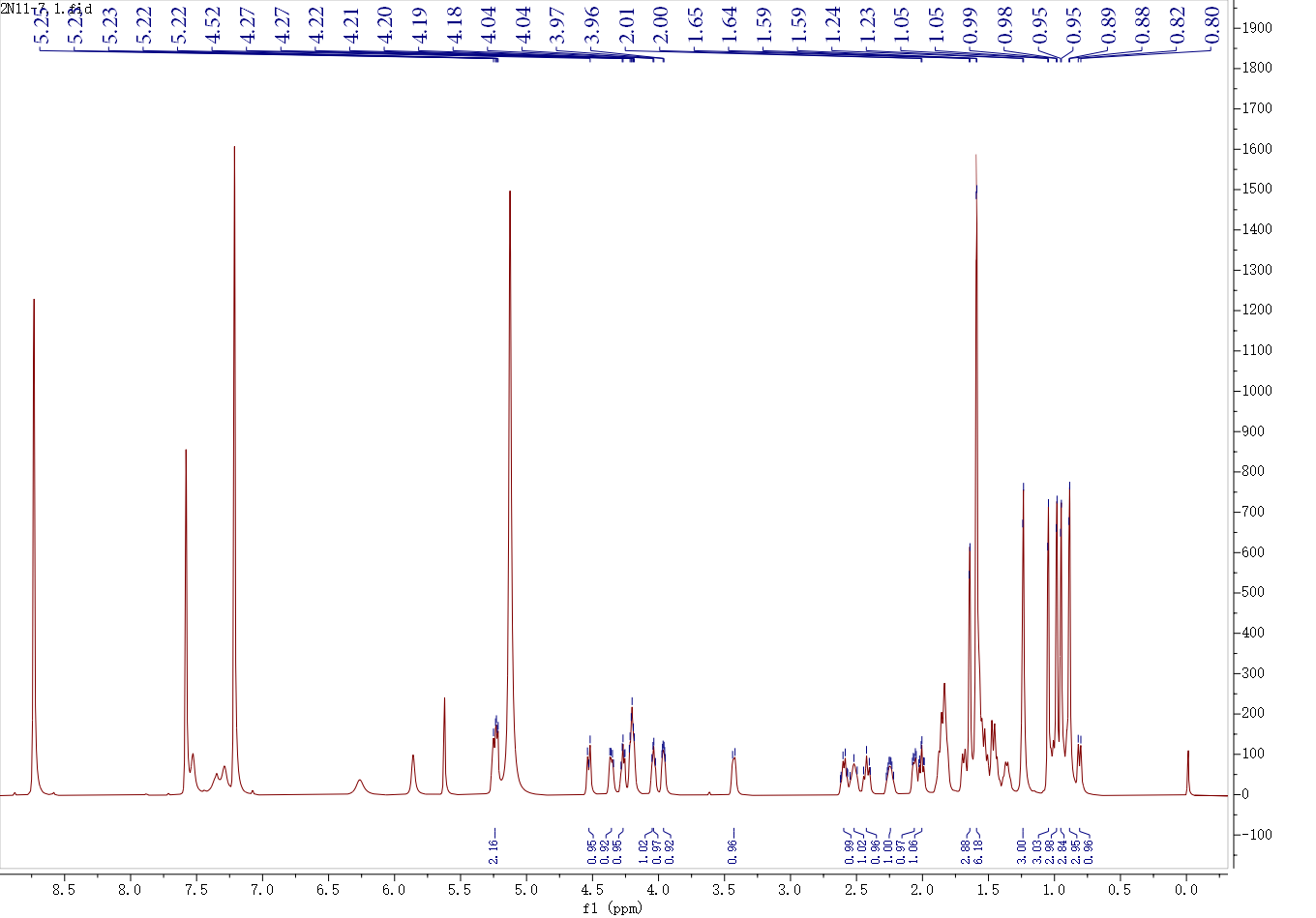


**Fig. S7** ^1^H-NMR and ^13^C-NMR spectra of compound **5**


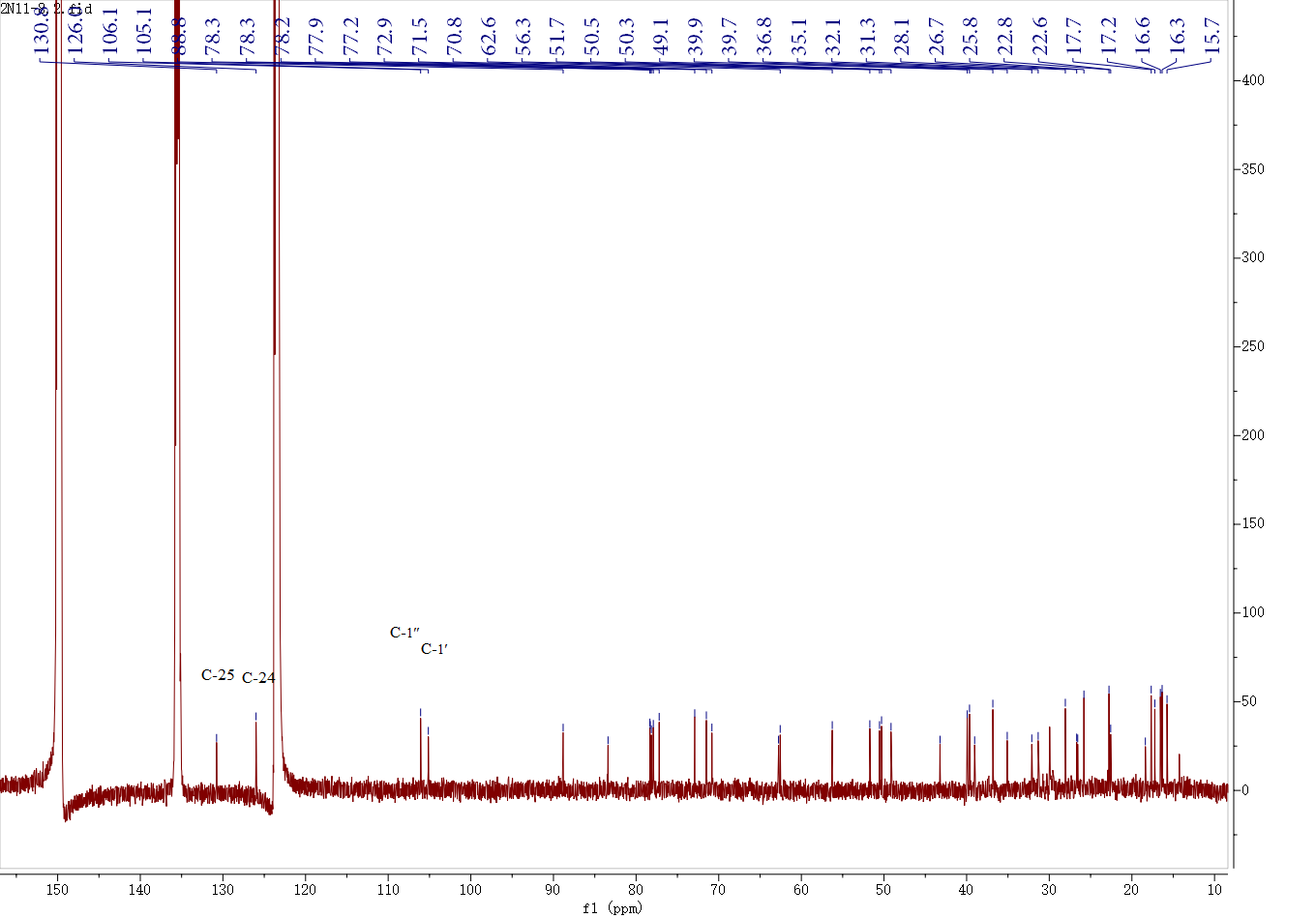

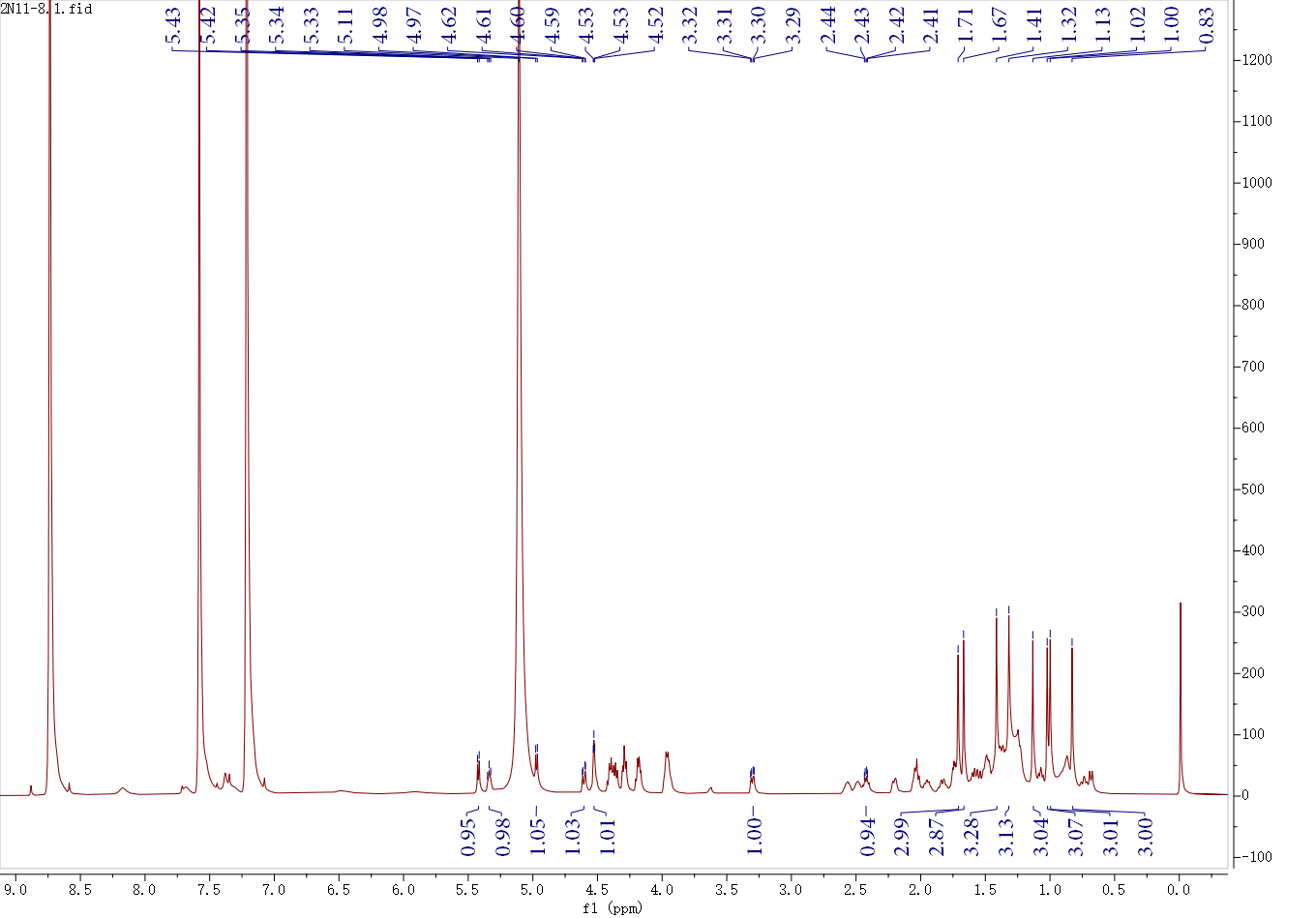


**Fig. S8** ^1^H-NMR and ^13^C-NMR spectra of compound **6**


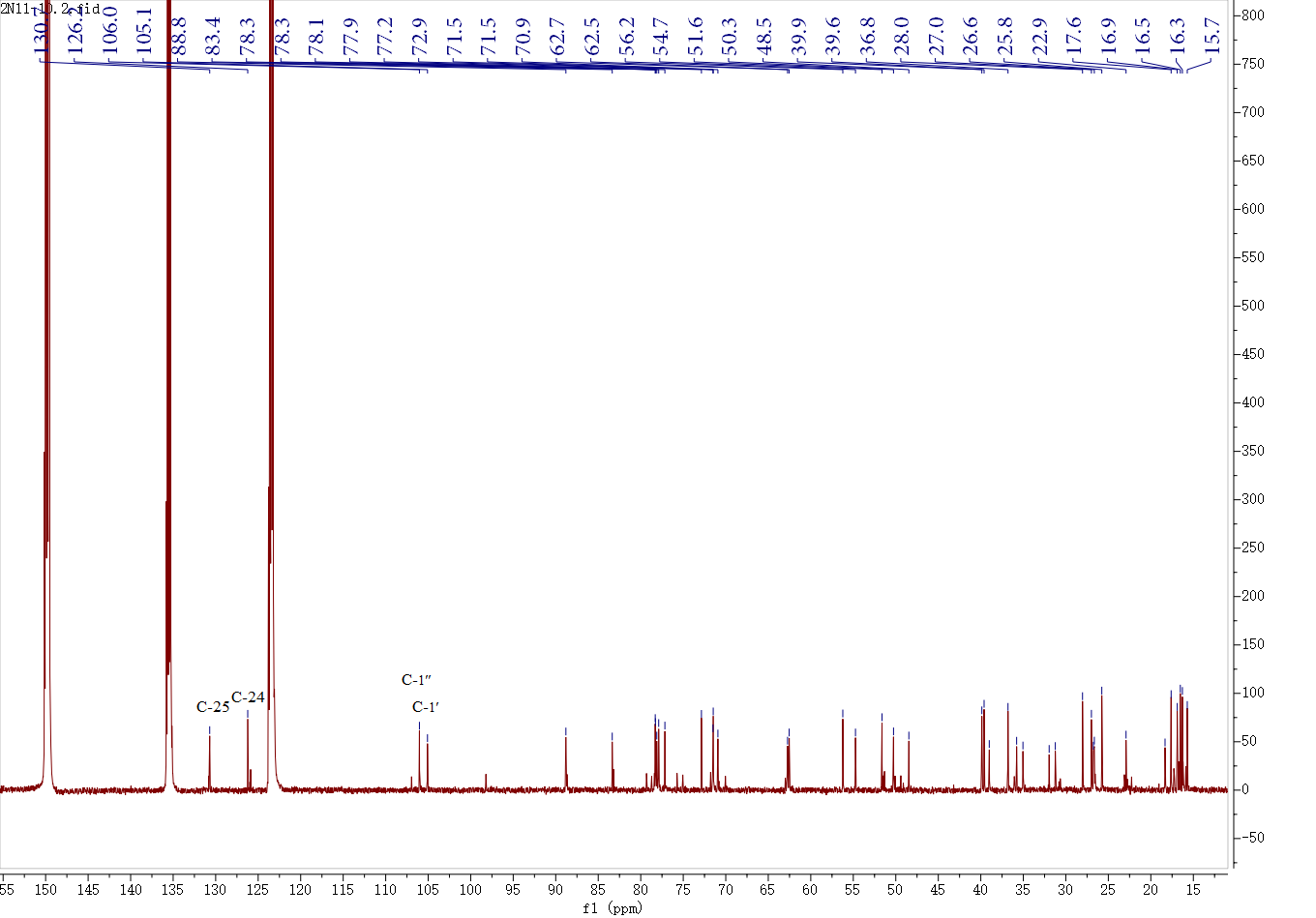

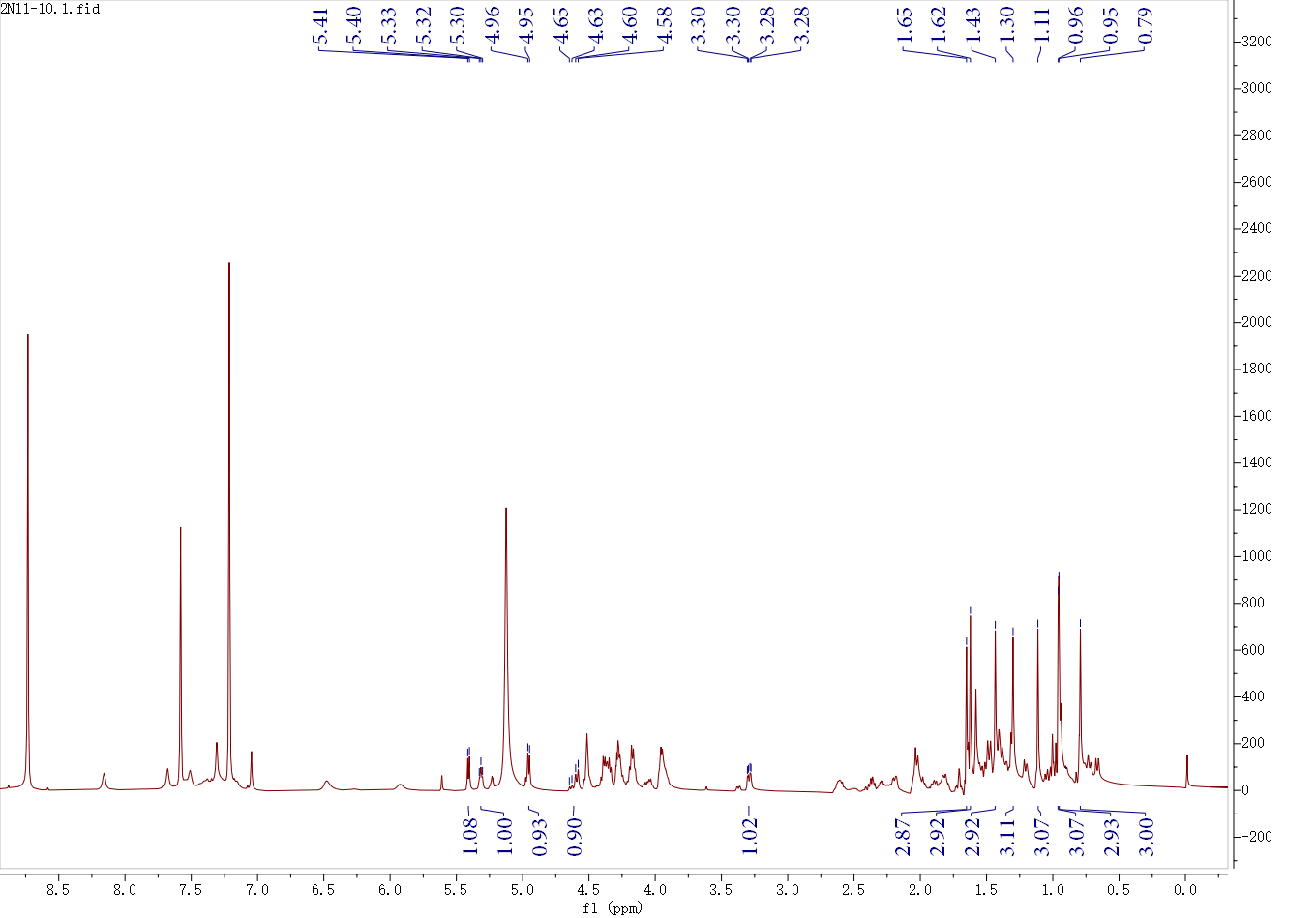


**Fig. S9** ^1^H-NMR and ^13^C-NMR spectra of compound **7**


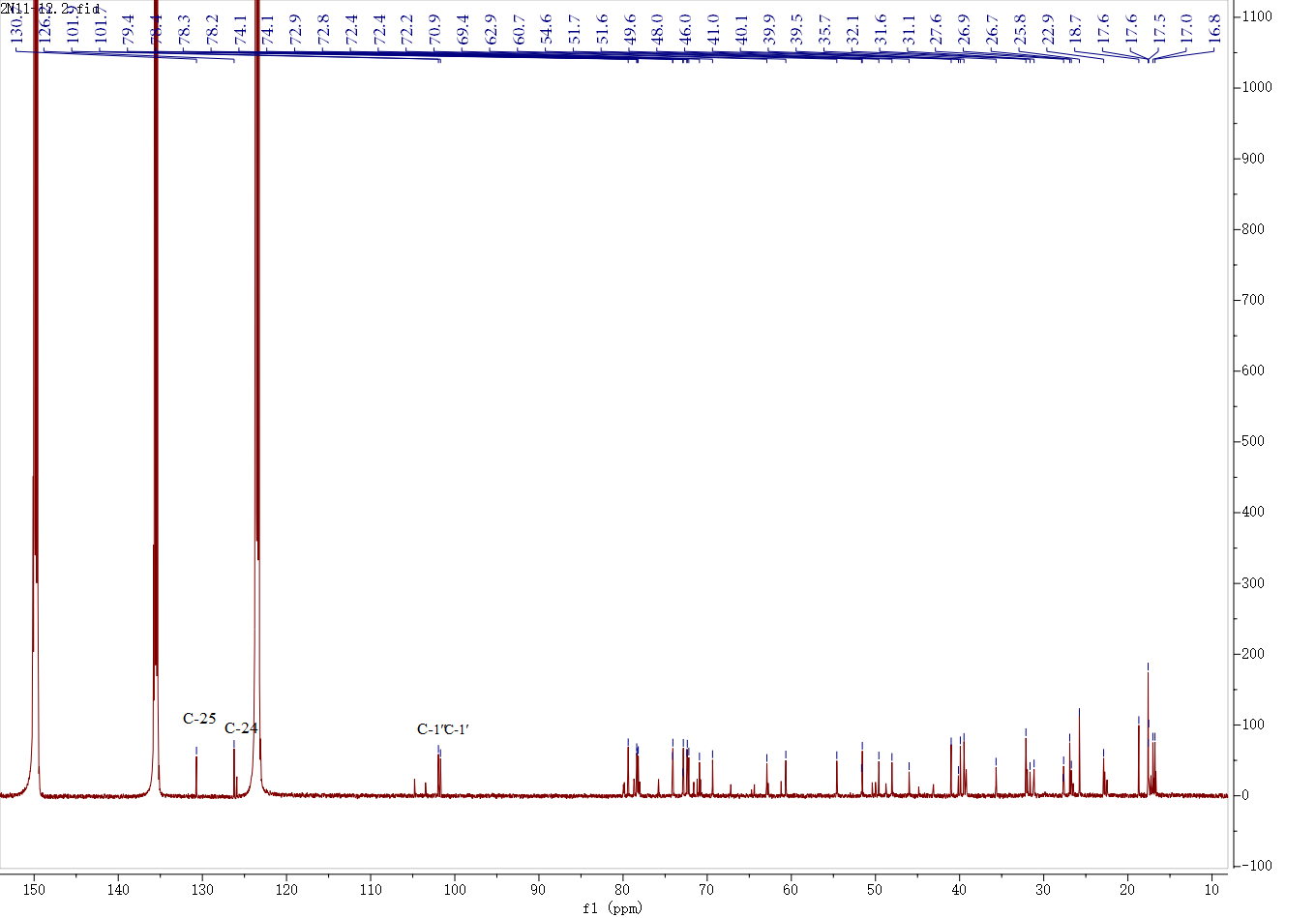

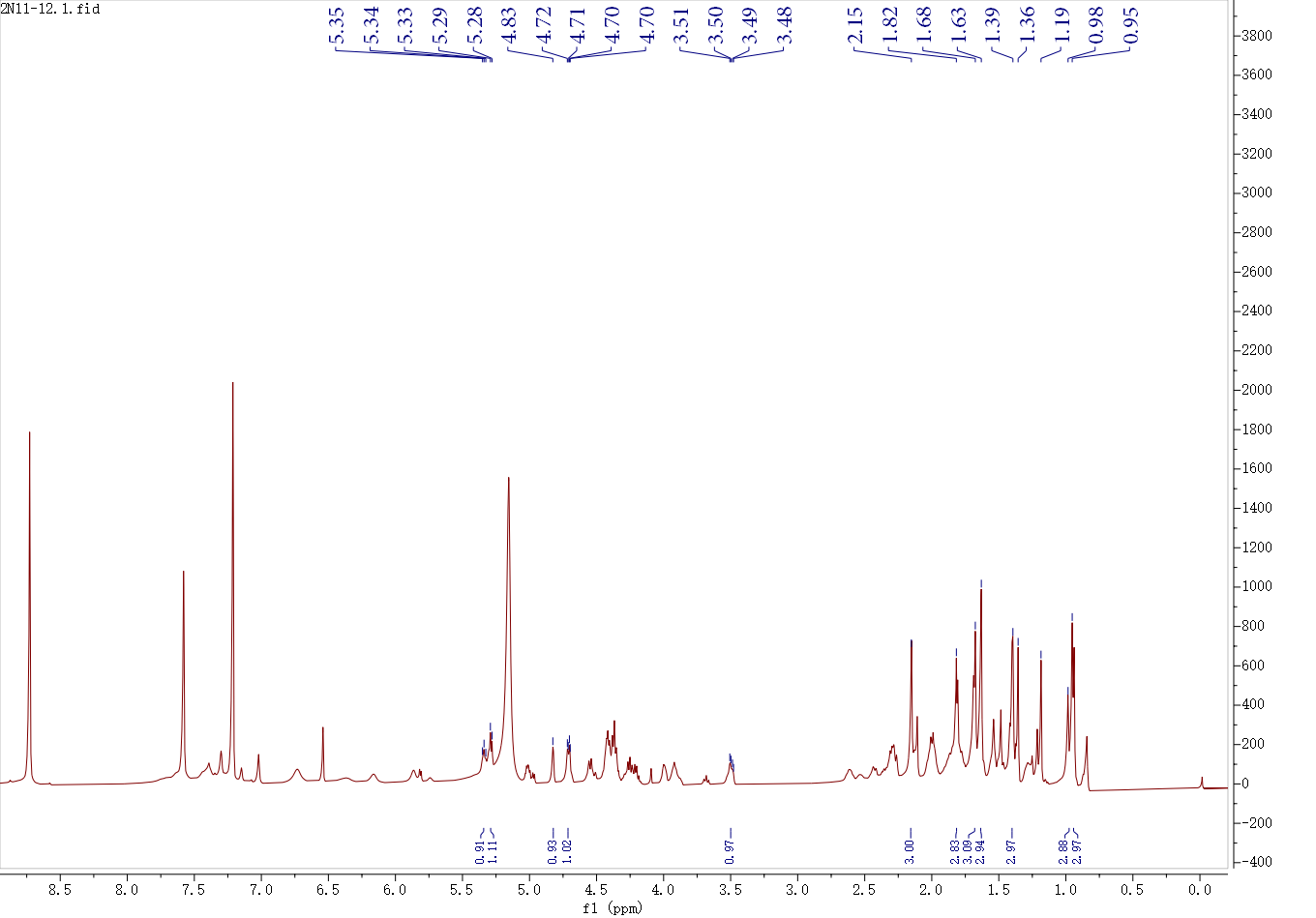


**Fig. S10** ^1^H-NMR and ^13^C-NMR spectra of compound **8**


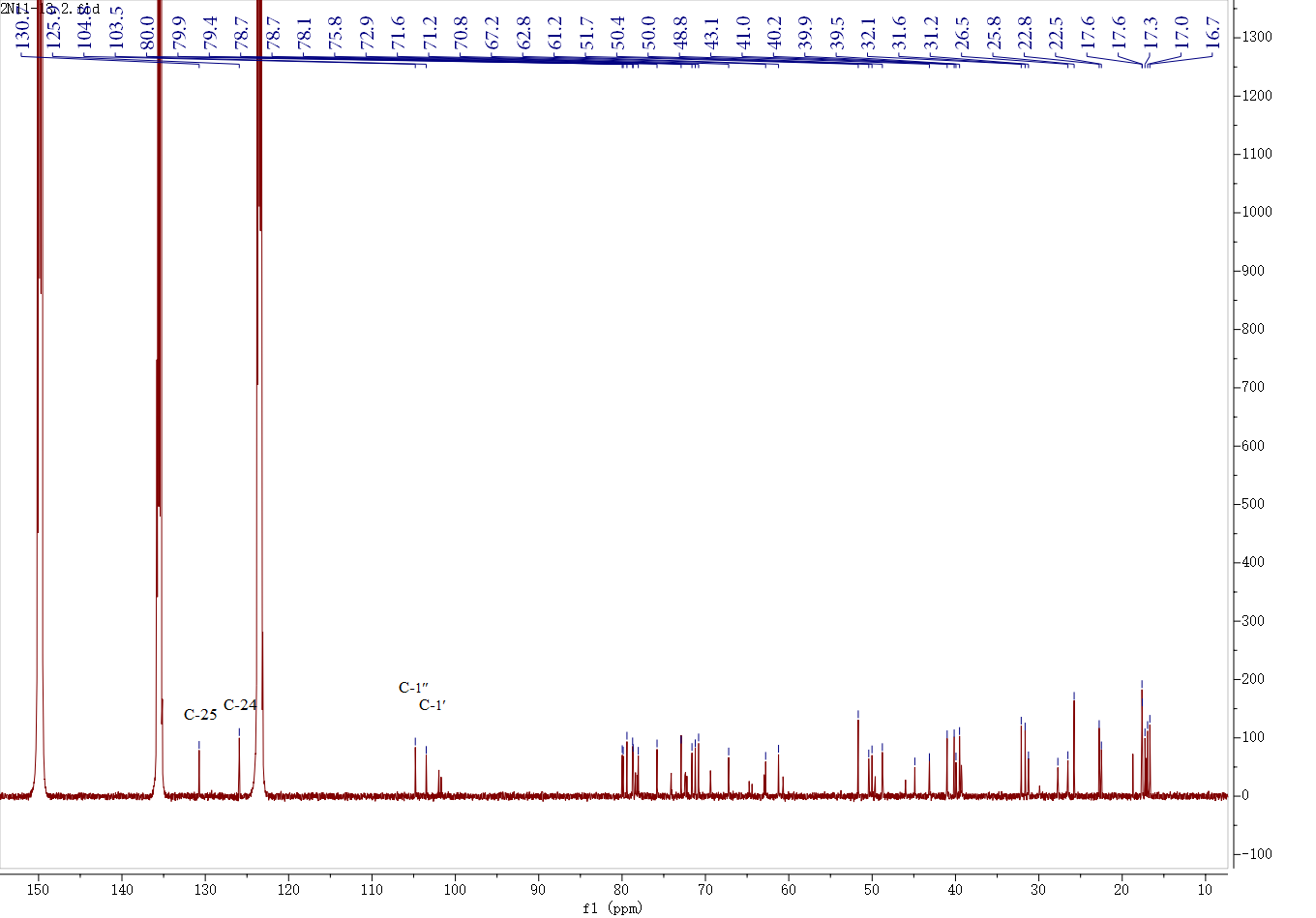

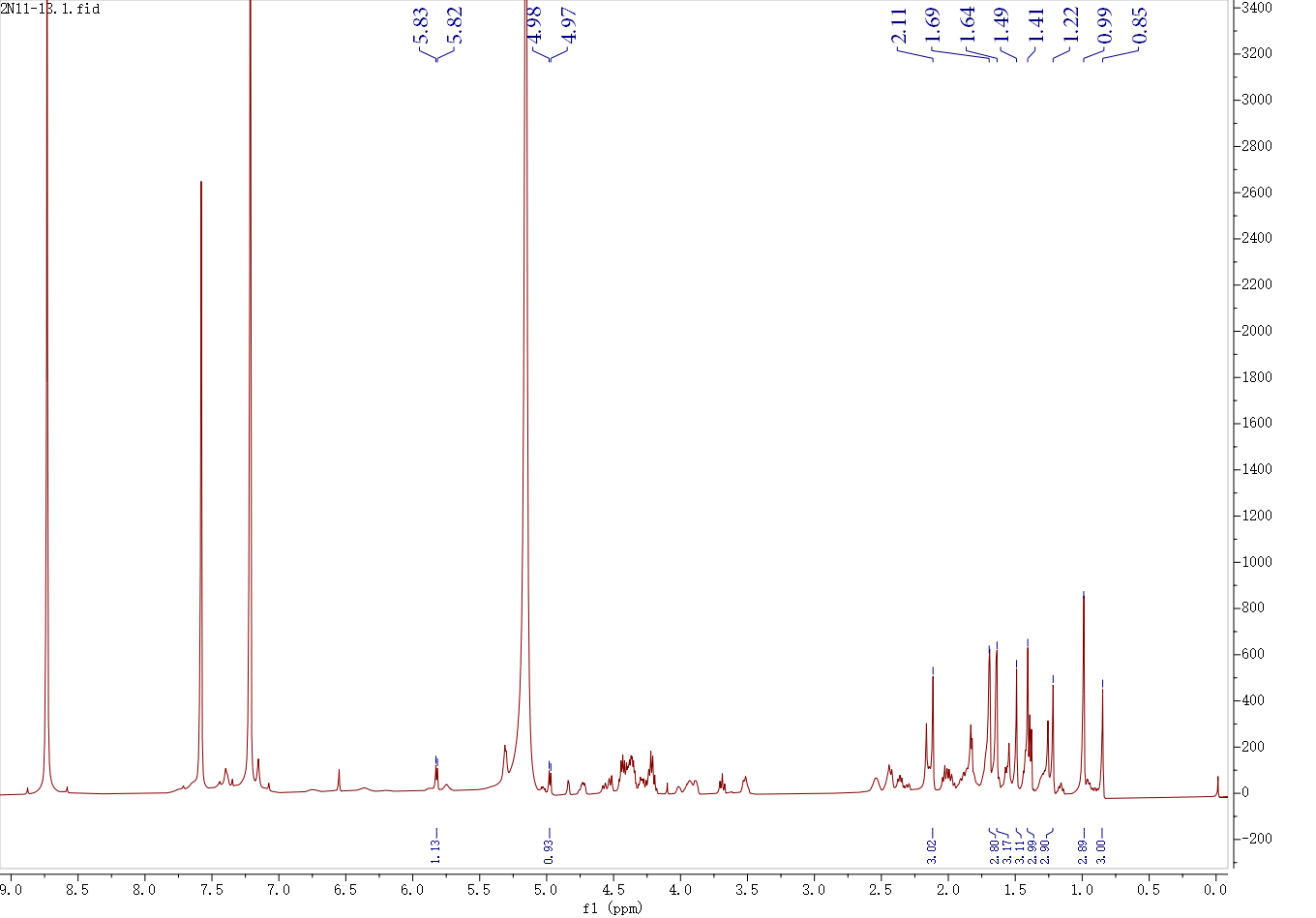


**Fig. S11** ^1^H-NMR and ^13^C-NMR spectra of compound **9**


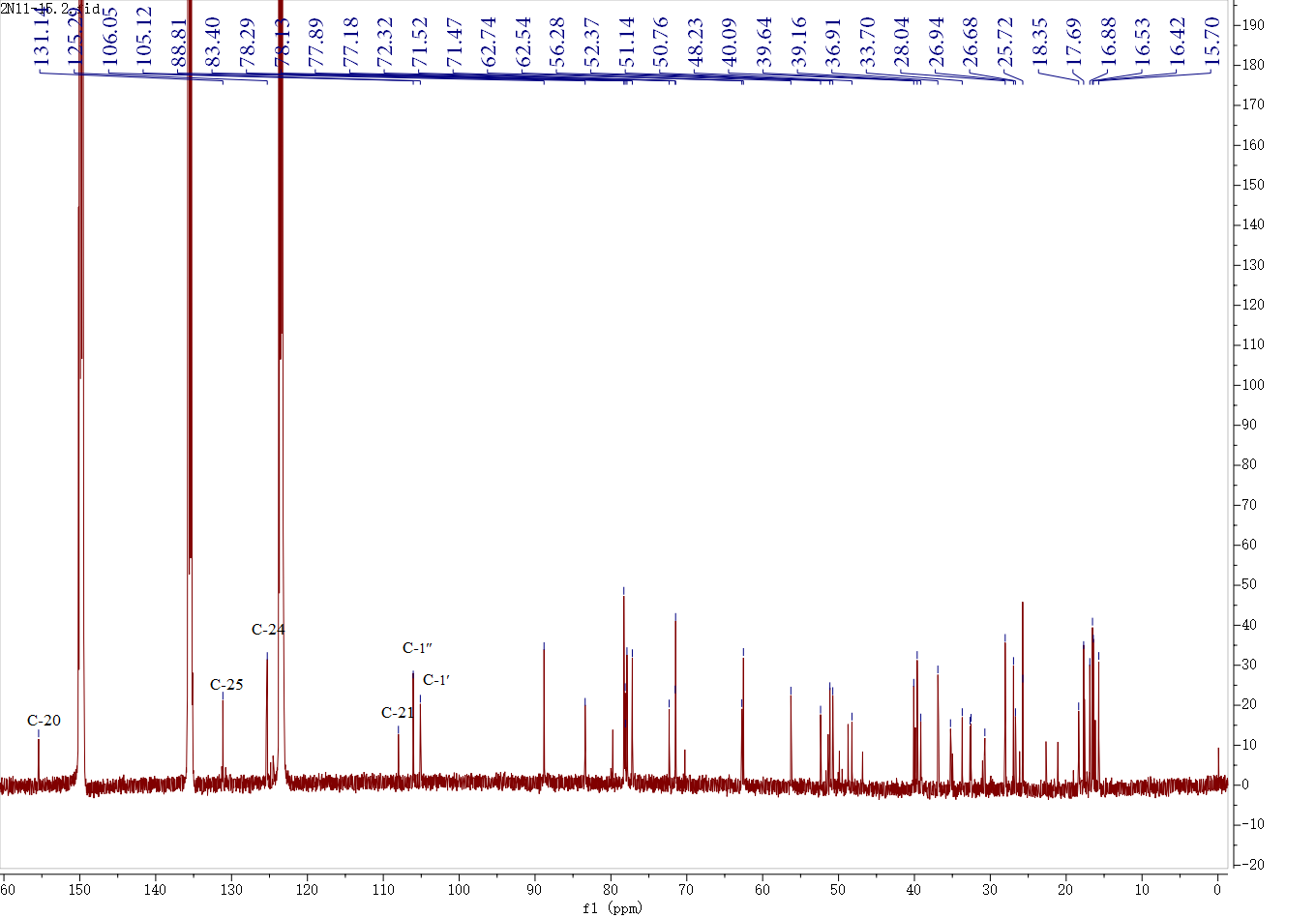

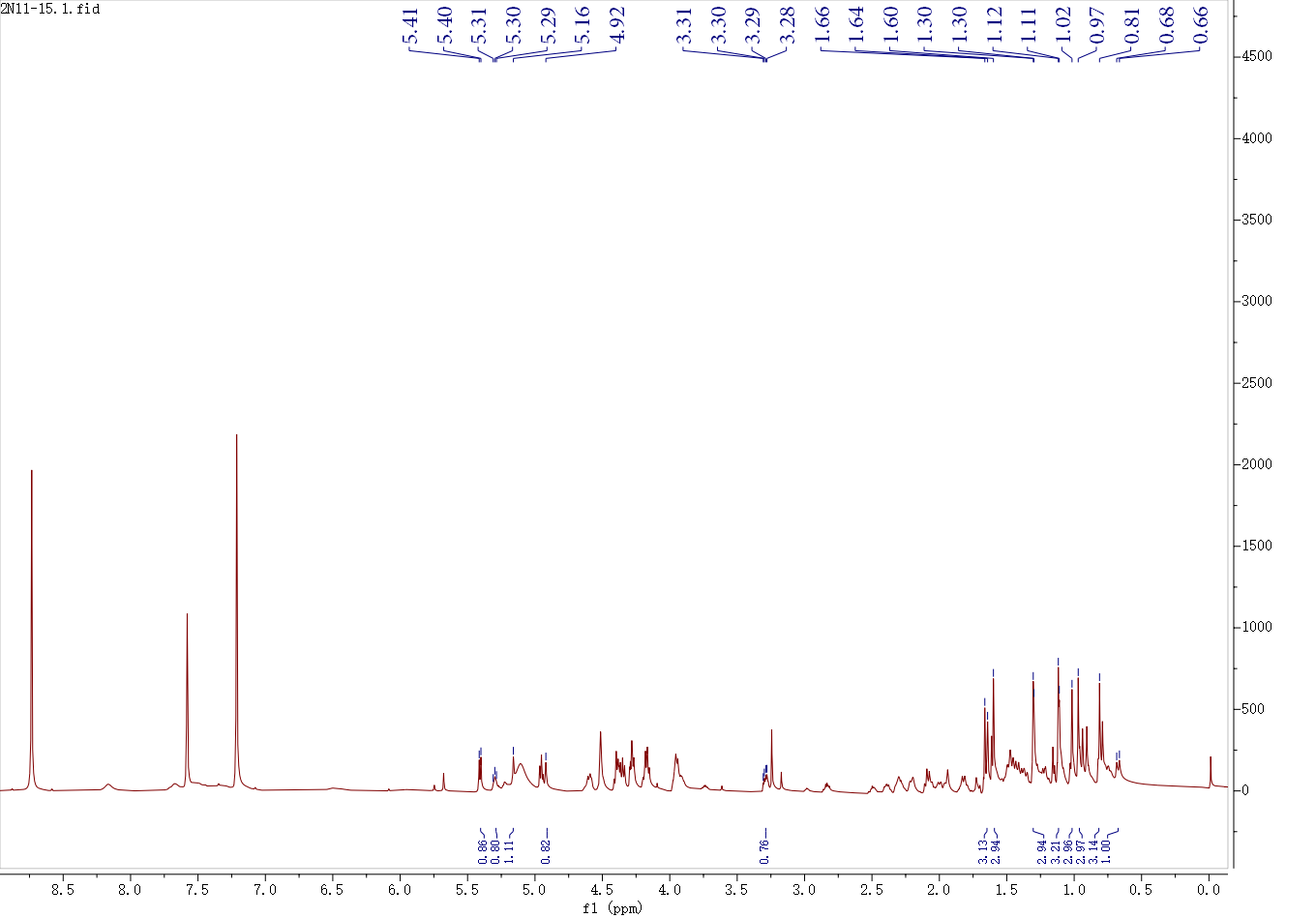


**Fig. S12** ^1^H-NMR and ^13^C-NMR spectra of compound **10**


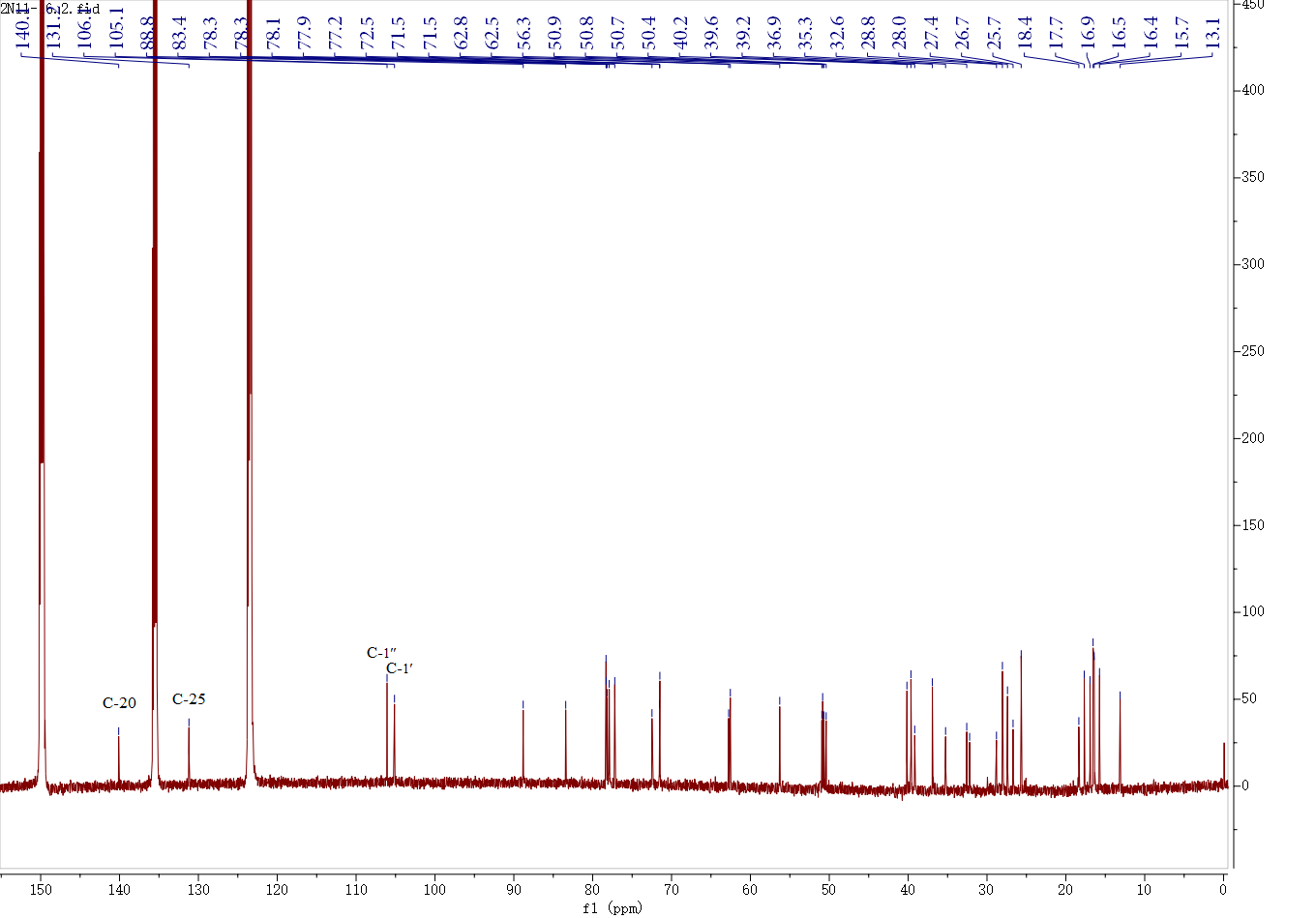

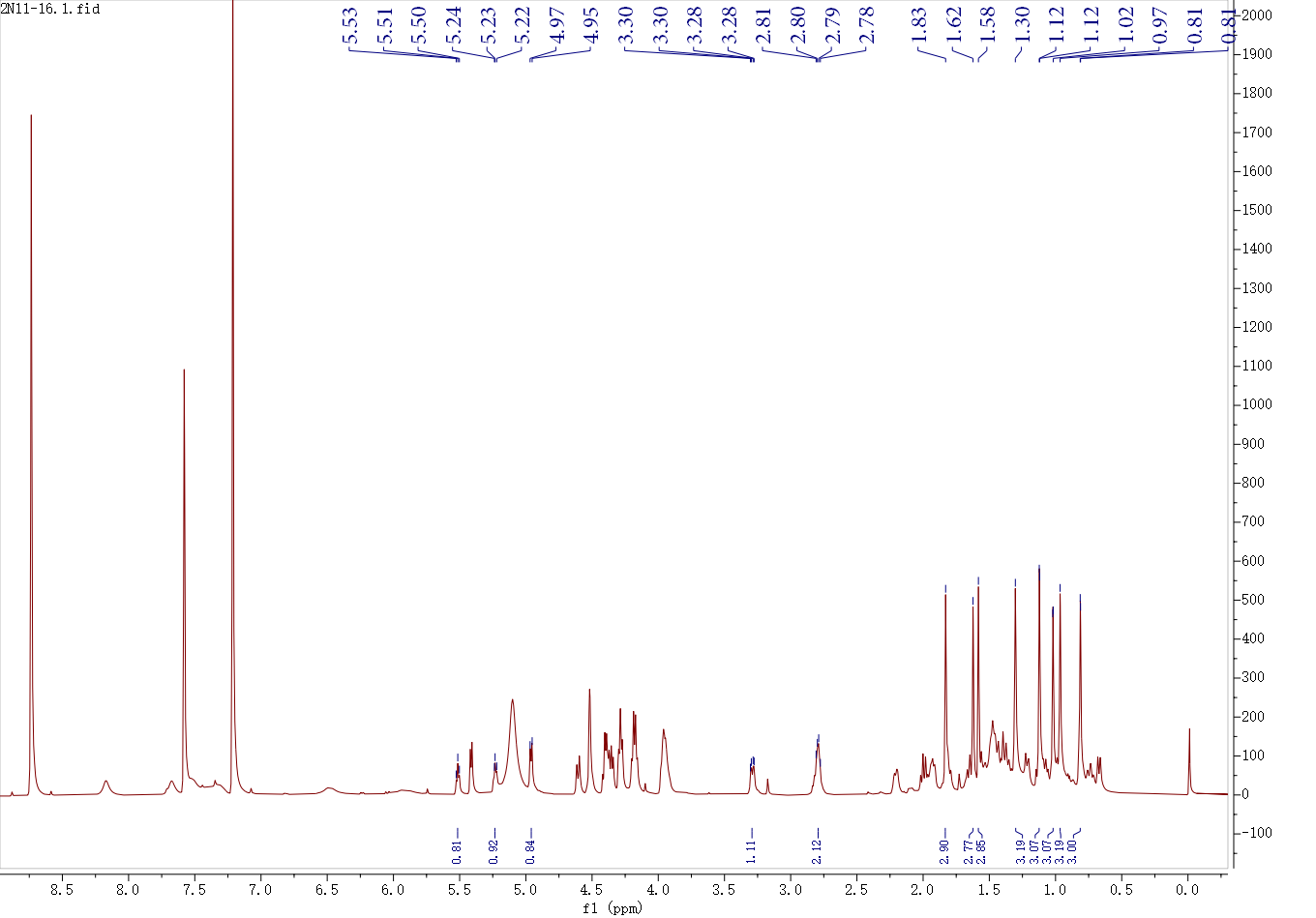


**Fig. S13** ^1^H-NMR and ^13^C-NMR spectra of compound **11**


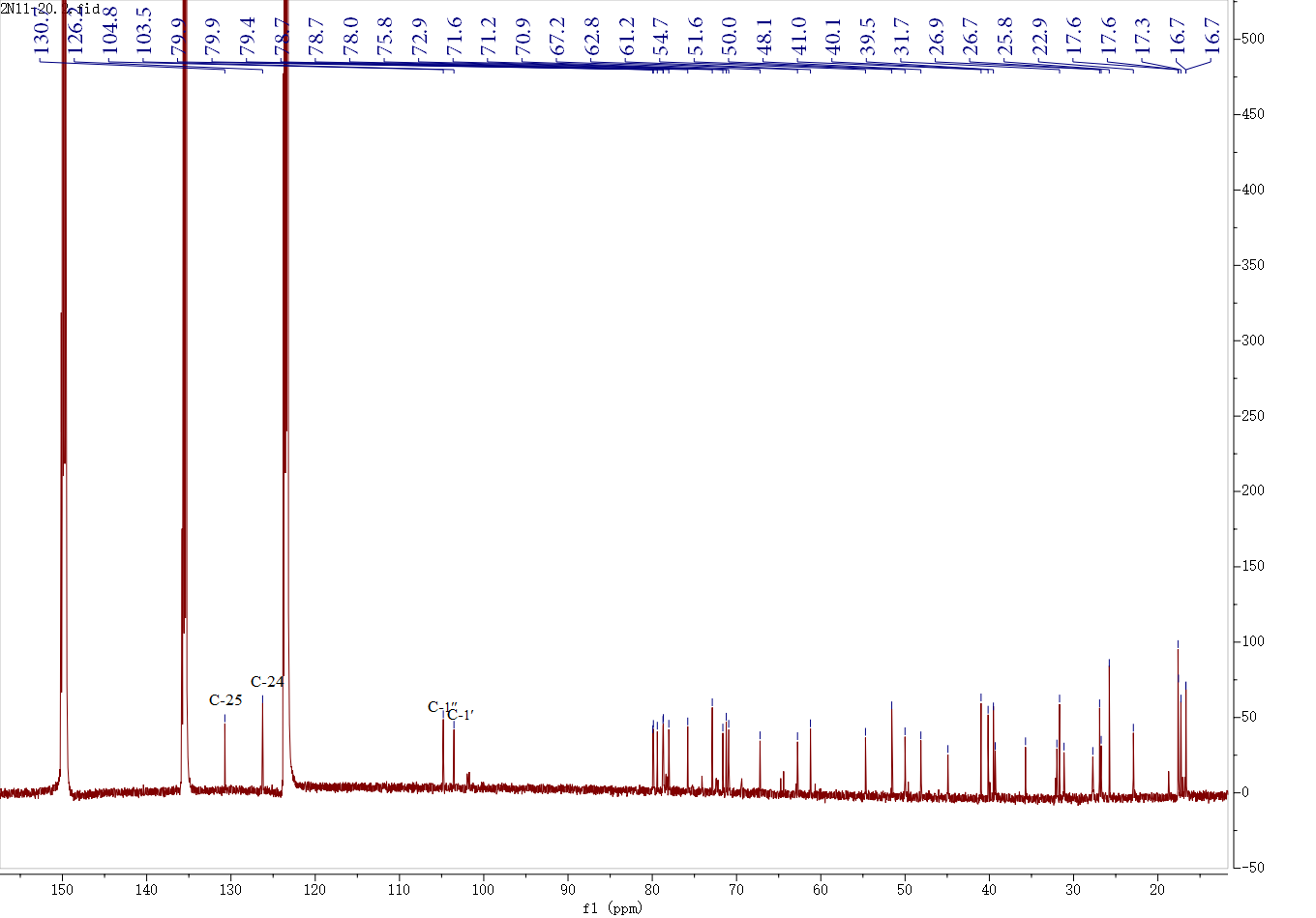

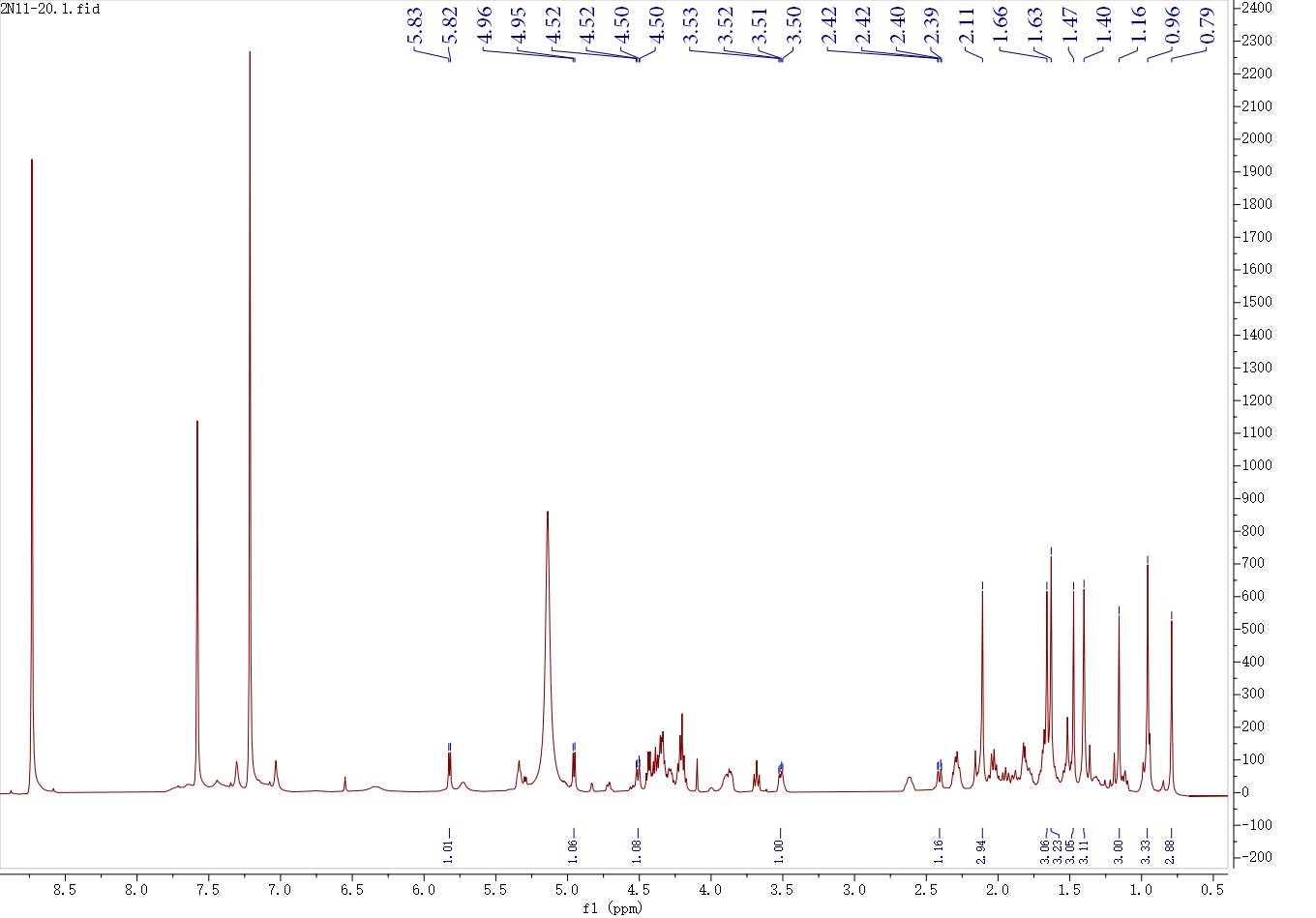


**Fig. S14** ^1^H-NMR and ^13^C-NMR spectra of compound **12**

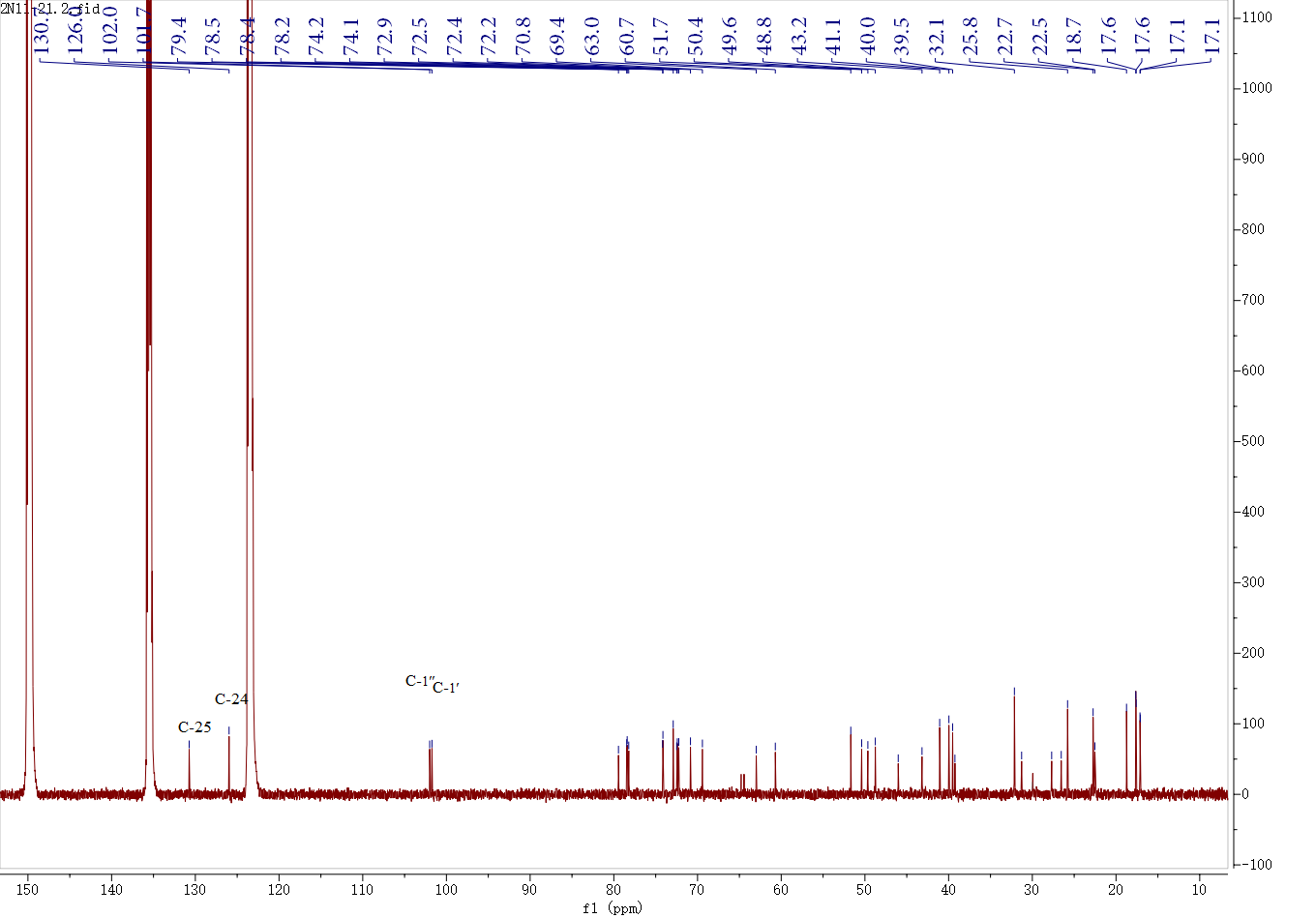
**Fig. S15** ^1^H-NMR and ^13^C-NMR spectra of compound **13**


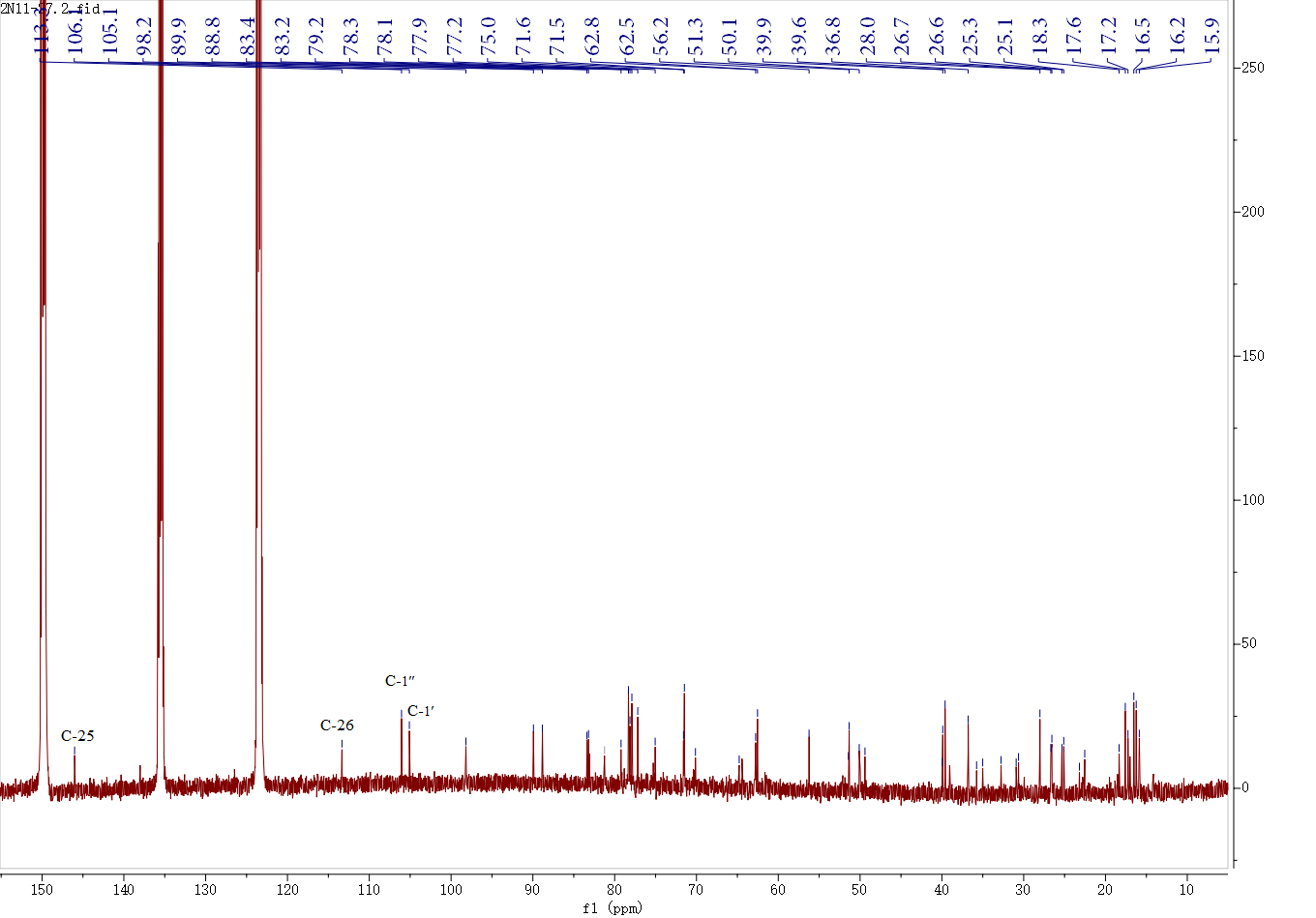

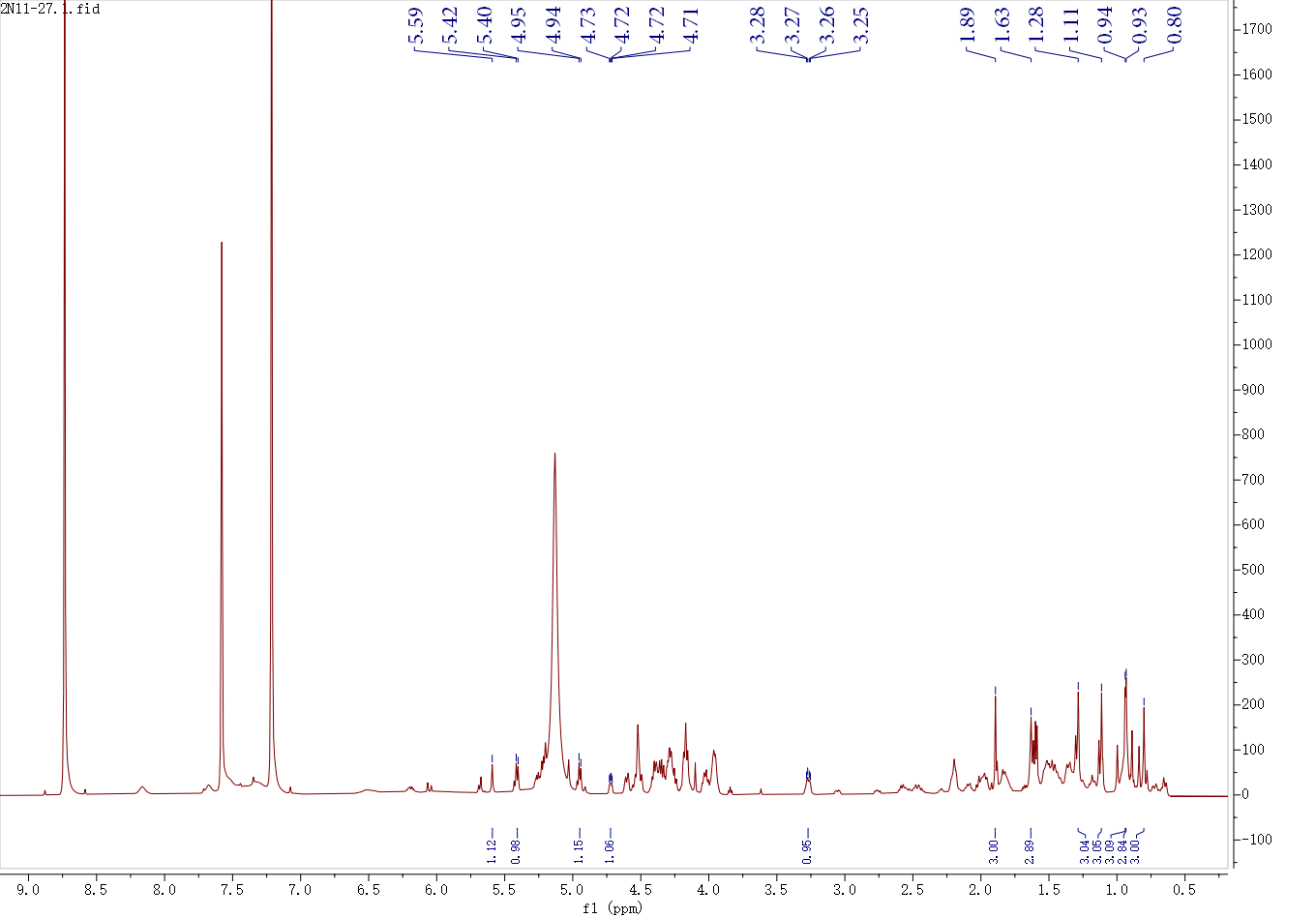


**Fig. S16** ^1^H-NMR and ^13^C-NMR spectra of compound **14**

**Fig. S17** Separation flow diagram of compounds **1**-**14**


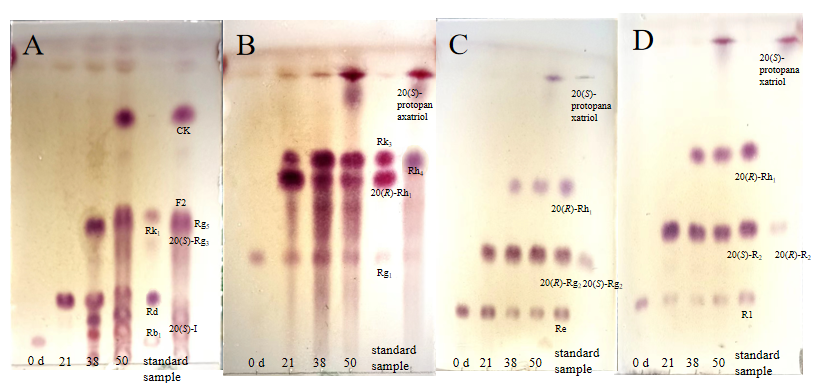


**Fig. S18** TLC analysis of transformation products at different times during the conversion process. A, B, C, D are the TLC analysis of transformation products of Rb_1_/Rd, Rg_1_, Re and R_1_, respectively.

**Table S1** ^1^H and ^13^C NMR spectral data of **1**-**2** (600 MHz, C_5_D_5_N, *δ* in ppm, *J* in Hz).

| **1** | | | **2** | | |
| --- | --- | --- | --- | --- | --- |
| NO. | *δ*_H_ (*J* Hz) | *δ*_C_ | NO. | *δ*_H_ (*J* Hz) | *δ*_C_ |
| 1 | - | 39.4 | 1 | - | 39.2 |
| 2 | - | 27.9 | 2 | - | 28.6 |
| 3 | 3.56 (dd, 11.7, 4.7 Hz) | 78.5 | 3 | 3.55 (d, 11.5, 4.2 Hz) | 78.3 |
| 4 | - | 40.4 | 4 | - | 40.1 |
| 5 | - | 61.4 | 5 | - | 61.1 |
| 6 | - | 80.0 | 6 | 4.44 (d, 11.5 Hz) | 79.8 |
| 7 | - | 45.2 | 7 | - | 45.0 |
| 8 | - | 41.2 | 8 | - | 41.0 |
| 9 | - | 50.6 | 9 | - | 50.3 |
| 10 | - | 39.6 | 10 | - | 39.4 |
| 11 | - | 32.7 | 11 | - | 32.2 |
| 12 | 3.91 (m) | 72.3 | 12 | 3.97 (m) | 72.3 |
| 13 | - | 52.0 | 13 | - | 50.3 |
| 14 | - | 51.1 | 14 | - | 50.1 |
| 15 | - | 32.4 | 15 | - | 32.0 |
| 16 | - | 30.7 | 16 | - | 27.2 |
| 17 | - | 48.3 | 17 | - | 50.6 |
| 18 | 1.24 (s) | 17.7 | 18 | 1.25 (s) | 17.4 |
| 19 | 1.05 (s) | 17.7 | 19 | 1.04 (s) | 17.1 |
| 20 | - | 155.3 | 20 | - | 139.8 |
| 21 | 5.13 (br s), 4.91 (br s) | 108.0 | 21 | 1.84 (s) | 12.9 |
| 22 | - | 33.6 | 22 | 5.45 (overlapped) | 123.0 |
| 23 | - | 26.9 | 23 | - | 27.6 |
| 24 | 5.29 (t, 6.8 Hz) | 125.3 | 24 | 5.19 (overlapped) | 125.4 |
| 25 | - | 131.1 | 25 | - | 131.0 |
| 26 | 1.67 (s) | 25.7 | 26 | 1.64 (s) | 25.4 |
| 27 | 1.64 (s) | 17.3 | 27 | 1.63 (s) | 17.4 |
| 28 | 2.11 (s) | 31.7 | 28 | 2.10 (s) | 31.4 |
| 29 | 1.60 (s) | 16.3 | 29 | 1.64 (s) | 16.1 |
| 30 | 0.83 (s) | 16.6 | 30 | 0.84 (s) | 16.5 |
| 1′ | 5.06 (d, 7.8 Hz) | 106.0 | 1′ | 5.05 (d, 7.8 Hz) | 105.7 |
| 2′ | - | 75.4 | 2′ | - | 75.2 |
| 3′ | - | 79.7 | 3′ | - | 79.3 |
| 4′ | - | 71.7 | 4′ | - | 71.4 |
| 5′ | - | 78.2 | 5′ | - | 77.9 |
| 6′ | - | 63.0 | 6′ | - | 62.7 |

**Table S2** ^1^H and ^13^C NMR spectral data of **3**-**4** (600 MHz, C_5_D_5_N, *δ* in ppm, *J* in Hz).

|  | **3** |  |  | **4** |  |
| --- | --- | --- | --- | --- | --- |
| NO. | *δ*_H_ (*J* Hz) | *δ*_C_ | NO. | *δ*_H_ (*J* Hz) | *δ*_C_ |
| 1 | - | 39.3 | 1 | - | 39.3 |
| 2 | - | 27.8 | 2 | - | 28.1 |
| 3 | - | 78.4 | 3 | 3.56 (dd, 11.6, 4.6 Hz) | 78.3 |
| 4 | - | 40.3 | 4 | - | 41.1 |
| 5 | - | 61.3 | 5 | - | 61.7 |
| 6 | 4.56 (m) | 80.0 | 6 | 4.42 (m) | 67.6 |
| 7 | - | 45.1 | 7 | - | 47.5 |
| 8 | - | 41.0 | 8 | - | 41.1 |
| 9 | - | 50.1 | 9 | - | 50.0 |
| 10 | - | 39.5 | 10 | - | 40.3 |
| 11 | - | 32.1 | 11 | - | 32.0 |
| 12 | - | 70.8 | 12 | 3.97 (m) | 71.0 |
| 13 | - | 48.8 | 13 | - | 48.2 |
| 14 | - | 51.6 | 14 | - | 51.6 |
| 15 | - | 31.2 | 15 | - | 31.3 |
| 16 | - | 26.5 | 16 | - | 26.8 |
| 17 | - | 50.4 | 17 | - | 54.7 |
| 18 | 1.24 (s) | 17.3 | 18 | 1.01 (s) | 17.5 |
| 19 | 1.06 (s) | 17.6 | 19 | 1.12 (s) | 17.4 |
| 20 |  | 72.9 | 20 | - | 72.9 |
| 21 | 1.40 (s) | 22.7 | 21 | 1.43 (s) | 27.0 |
| 22 | - | 43.1 | 22 | - | 35.8 |
| 23 | - | 22.5 | 23 | - | 22.9 |
| 24 | 5.30 (t, 6.8 Hz) | 125.9 | 24 | 5.33 (t, 7.2 Hz) | 126.2 |
| 25 | - | 130.7 | 25 |  | 130.8 |
| 26 | 1.69 (s) | 25.8 | 26 | 1.66 (s) | 25.8 |
| 27 | 1.64 (s) | 17.6 | 27 | 1.63 (s) | 17.6 |
| 28 | 2.10 (s) | 31.6 | 28 | 2.03 (s) | 31.9 |
| 29 | 1.63 (s) | 16.3 | 29 | 1.47 (s) | 16.5 |
| 30 | 0.86 (s) | 16.9 | 30 | 0.97 (s) | 17.0 |
| 1′ | 5.06 (d, 7.9 Hz) | 105.9 |  |  |  |
| 2′ | - | 75.4 |  |  |  |
| 3′ | - | 79.6 |  |  |  |
| 4′ | - | 71.7 |  |  |  |
| 5′ | - | 78.1 |  |  |  |
| 6′ | - | 62.9 |  |  |  |

**Table S3** ^1^H and ^13^C NMR spectral data of **5**-**6** (600 MHz, C_5_D_5_N, *δ* in ppm, *J* in Hz).

|  | **5** |  |  | **6** |  |
| --- | --- | --- | --- | --- | --- |
| NO. | *δ*_H_ (*J* Hz) | *δ*_C_ | NO. | *δ*_H_ (*J* Hz) | *δ*_C_ |
| 1 | - | 39.3 | 1 | - | 39.0 |
| 2 | - | 28.2 | 2 | - | 26.6 |
| 3 | 3.43 (d, 10.9 Hz) | 77.9 | 3 | 3.30 (dd, 11.8, 4.5 Hz) | 88.8 |
| 4 | - | 39.5 | 4 | - | 39.7 |
| 5 | - | 56.3 | 5 | - | 56.3 |
| 6 | - | 18.7 | 6 | - | 18.4 |
| 7 | - | 35.1 | 7 | - | 35.1 |
| 8 | - | 40.0 | 8 | - | 39.9 |
| 9 | - | 50.2 | 9 | - | 50.3 |
| 10 | - | 37.3 | 10 | - | 36.8 |
| 11 | - | 30.7 | 11 | - | 32.1 |
| 12 | - | 70.1 | 12 | - | 70.8 |
| 13 | - | 49.4 | 13 | - | 49.1 |
| 14 | - | 51.3 | 14 | - | 51.7 |
| 15 | - | 30.9 | 15 | - | 31.3 |
| 16 | - | 26.6 | 16 | - | 26.7 |
| 17 | - | 51.5 | 17 | - | 50.5 |
| 18 | 0.95 (s) | 16.3 | 18 | 1.02 (s) | 15.7 |
| 19 | 0.89 (s) | 15.9 | 19 | 0.83 (s) | 16.3 |
| 20 | - | 83.2 | 20 | - | 72.9 |
| 21 | 1.64 (s) | 22.3 | 21 | 1.41 (s) | 22.8 |
| 22 | - | 36.1 | 22 | - | 43.2 |
| 23 | - | 23.1 | 23 | - | 22.6 |
| 24 | 5.23 (t, 7.3 Hz) | 125.9 | 24 | 5.33 (t, 7.5 Hz) | 125.9 |
| 25 | - | 130.8 | 25 | - | 130.8 |
| 26 | 1.59 (s) | 25.7 | 26 | 1.71 (s) | 25.8 |
| 27 | 1.59 (s) | 17.7 | 27 | 1.67 (s) | 17.7 |
| 28 | 1.24 (s) | 28.6 | 28 | 1.32 (s) | 28.2 |
| 29 | 1.05 (s) | 16.3 | 29 | 1.13 (s) | 16.6 |
| 30 | 0.98 (s) | 17.3 | 30 | 1.00 (s) | 17.2 |
| 1′ | 5.22 (d, 7.3 Hz) | 98.2 | 1′ | 4.97 (d, 7.6 Hz) | 105.1 |
| 2′ | - | 75.1 | 2′ | - | 83.4 |
| 3′ | - | 79.3 | 3′ | - | 77.9 |
| 4′ | - | 71.5 | 4′ | - | 71.5 |
| 5′ | - | 78.3 | 5′ | - | 78.2 |
| 6′ | - | 62.8 | 6′ | - | 62.8 |
|  |  |  | 1′′ | 5.42 (d, 7.7 Hz) | 106.1 |
|  |  |  | 2′′ | - | 77.2 |
|  |  |  | 3′′ | - | 78.3 |
|  |  |  | 4′′ | - | 71.5 |
|  |  |  | 5′′ | - | 78.3 |
|  |  |  | 6′′ | - | 62.6 |

**Table S4** ^1^H and ^13^C NMR spectral data of **7**-**8** (600 MHz, C_5_D_5_N, *δ* in ppm, *J* in Hz).

|  | **7** |  |  | **8** |  |
| --- | --- | --- | --- | --- | --- |
| NO. | *δ*_H_ (*J* Hz) | *δ*_C_ | NO. | *δ*_H_ (*J* Hz) | *δ*_C_ |
| 1 | - | 39.0 | 1 | - | 39.9 |
| 2 | - | 26.6 | 2 | - | 27.6 |
| 3 | 3.29 (dd, 11.8, 4.5 Hz) | 88.8 | 3 | 3.49 (dd, 11.9, 4.8 Hz) | 78.3 |
| 4 | - | 39.7 | 4 | - | 40.1 |
| 5 | - | 56.3 | 5 | - | 60.7 |
| 6 | - | 18.4 | 6 | 4.72 (m) | 74.1 |
| 7 | - | 35.1 | 7 | - | 46.0 |
| 8 | - | 39.9 | 8 | - | 41.0 |
| 9 | - | 50.3 | 9 | - | 49.6 |
| 10 | - | 36.8 | 10 | - | 39.5 |
| 11 | - | 32.1 | 11 | - | 31.6 |
| 12 | 3.95 (m) | 70.8 | 12 | - | 70.9 |
| 13 | - | 48.5 | 13 | - | 48.0 |
| 14 | - | 51.7 | 14 | - | 51.6 |
| 15 | - | 31.3 | 15 | - | 31.1 |
| 16 | - | 26.7 | 16 | - | 26.9 |
| 17 | - | 54.8 | 17 | - | 54.6 |
| 18 | 0.96 (s) | 15.7 | 18 | 1.19 (s) | 17.5 |
| 19 | 0.79 (s) | 16.3 | 19 | 0.98 (s) | 17.0 |
| 20 | - | 72.9 | 20 | - | 72.9 |
| 21 | 1.43 (s) | 28.1 | 21 | 1.39 (s) | 22.7 |
| 22 | - | 35.8 | 22 | - | 35.7 |
| 23 | - | 22.9 | 23 | - | 22.9 |
| 24 | 5.32 (t, 7.4 Hz) | 126.0 | 24 | 5.34 (t, 7.0 Hz) | 126.2 |
| 25 | - | 130.8 | 25 | - | 130.7 |
| 26 | 1.65 (s) | 25.8 | 26 | 1.68 (s) | 25.8 |
| 27 | 1.62 (s) | 17.2 | 27 | 1.63 (s) | 17.6 |
| 28 | 1.30 (s) | 27.0 | 28 | 2.15 (s) | 27.7 |
| 29 | 1.11 (s) | 16.6 | 29 | 1.36 (s) | 16.8 |
| 30 | 0.95 (s) | 17.7 | 30 | 0.95 (s) | 17.6 |
| 1′ | 4.95 (d, 7.9 Hz) | 105.1 | 1′ | 5.29 (d, 7.3 Hz) | 101.9 |
| 2′ | - | 83.4 | 2′ | - | 78.2 |
| 3′ | - | 77.9 | 3′ | - | 79.4 |
| 4′ | - | 71.5 | 4′ | - | 72.8 |
| 5′ | - | 78.2 | 5′ | - | 78.4 |
| 6′ | - | 62.8 | 6′ | - | 62.9 |
| 1′′ | 5.41 (d, 7.6 Hz) | 106.1 | 1′′ | 6.54 (s) | 101.7 |
| 2′′ | - | 77.2 | 2′′ | - | 72.4 |
| 3′′ | - | 78.3 | 3′′ | - | 72.2 |
| 4′′ | - | 71.5 | 4′′ | - | 74.1 |
| 5′′ | - | 78.3 | 5′′ | - | 69.4 |
| 6′′ | - | 62.6 | 6′′ | 1.82 (d, 7.0 Hz) | 18.7 |

**Table S5** ^1^H and ^13^C NMR spectral data of **9**-**10** (600 MHz, C_5_D_5_N, *δ* in ppm, *J* in Hz).

|  | **9** |  |  | **10** |  |
| --- | --- | --- | --- | --- | --- |
| NO. | *δ*_H_ (*J* Hz) | *δ*_C_ | NO. | *δ*_H_ (*J* Hz) | *δ*_C_ |
| 1 | - | 39.5 | 1 | 4.95 (t, 7.4 Hz) | 39.2 |
| 2 | - | 27.7 | 2 | - | 26.7 |
| 3 | - | 78.7 | 3 | 3.29 (dd, 11.6, 4.9 Hz) | 88.8 |
| 4 | - | 40.2 | 4 | - | 39.6 |
| 5 | - | 61.2 | 5 | - | 56.3 |
| 6 | - | 79.4 | 6 | - | 18.4 |
| 7 | - | 44.9 | 7 | - | 35.2 |
| 8 | - | 41.0 | 8 | - | 40.1 |
| 9 | - | 50.0 | 9 | - | 48.2 |
| 10 | - | 39.9 | 10 | - | 36.9 |
| 11 | - | 32.1 | 11 | - | 32.5 |
| 12 | - | 70.8 | 12 | 3.95 (m) | 72.3 |
| 13 | - | 48.8 | 13 | - | 52.4 |
| 14 | - | 51.7 | 14 | - | 51.1 |
| 15 | - | 31.2 | 15 | - | 32.7 |
| 16 | - | 26.5 | 16 | - | 30.7 |
| 17 | - | 50.4 | 17 | - | 50.8 |
| 18 | 1.22 (s) | 17.3 | 18 | 1.65 (s) | 16.4 |
| 19 | 0.99 (s) | 17.6 | 19 | 0.81 (s) | 15.7 |
| 20 | - | 72.9 | 20 | - | 155.4 |
| 21 | 1.41 (s) | 22.8 | 21 | 5.16 (br s) 4.92 (br s) | 108.0 |
| 22 | - | 43.1 | 22 | - | 33.7 |
| 23 | - | 22.5 | 23 | - | 26.9 |
| 24 | - | 125.9 | 24 | 5.23 (br t, 6.7 Hz) | 125.3 |
| 25 | - | 130.7 | 25 | - | 131.1 |
| 26 | 1.69 (s) | 25.8 | 26 | 1.60 (s) | 25.7 |
| 27 | 1.64 (s) | 17.6 | 27 | 1.30 (s) | 17.7 |
| 28 | 2.11 (s) | 31.6 | 28 | 1.12 (s) | 28.0 |
| 29 | 1.49 (s) | 16.7 | 29 | 1.02 (s) | 16.5 |
| 30 | 0.85 (s) | 17.0 | 30 | 0.97 (s) | 16.9 |
| 1′ | 4.98 (d, 7.3 Hz) | 103.5 | 1′ | - | 105.1 |
| 2′ | - | 80.0 | 2′ | - | 83.4 |
| 3′ | - | 78.1 | 3′ | - | 78.1 |
| 4′ | - | 71.6 | 4′ | - | 71.5 |
| 5′ | - | 79.9 | 5′ | - | 77.9 |
| 6′ | - | 62.8 | 6′ | - | 62.5 |
| 1′′ | 5.82 (d, 7.1 Hz) | 104.8 | 1′′ | 5.29 (d, 7.3 Hz) | 106.1 |
| 2′′ | - | 75.8 | 2′′ | - | 77.2 |
| 3′′ | - | 78.7 | 3′′ | - | 78.3 |
| 4′′ | - | 71.2 | 4′′ | - | 71.5 |
| 5′′ | - | 67.2 | 5′′ | - | 78.1 |
|  |  |  | 6′′ | - | 62.7 |

**Table S6** ^1^H and ^13^C NMR spectral data of **11**-**12** (600 MHz, C_5_D_5_N, *δ* in ppm, *J* in Hz).

|  | **11** |  |  | **12** |  |
| --- | --- | --- | --- | --- | --- |
| NO. | *δ*_H_ (*J* Hz) | *δ*_C_ | NO. | *δ*_H_ (*J* Hz) | *δ*_C_ |
| 1 | - | 39.2 | 1 | - | 39.3 |
| 2 | - | 28.0 | 2 | - | 26.9 |
| 3 | 3.30 (dd, 11.6, 4.0Hz) | 88.8 | 3 | 3.51 (dd, 11.5, 4.8 Hz) | 78.7 |
| 4 | - | 40.2 | 4 | - | 40.1 |
| 5 | - | 56.3 | 5 | - | 61.2 |
| 6 | - | 18.4 | 6 | - | 79.9 |
| 7 | - | 35.3 | 7 | - | 44.9 |
| 8 | - | 39.6 | 8 | - | 41.0 |
| 9 | - | 50.7 | 9 | - | 50.0 |
| 10 | - | 36.9 | 10 | - | 39.5 |
| 11 | - | 32.2 | 11 | - | 32.0 |
| 12 | 3.96 (m) | 72.5 | 12 | - | 70.9 |
| 13 | - | 50.4 | 13 | - | 48.1 |
| 14 | - | 50.9 | 14 | - | 51.6 |
| 15 | - | 32.6 | 15 | - | 31.1 |
| 16 | - | 26.7 | 16 | - | 27.7 |
| 17 | - | 50.8 | 17 | - | 54.7 |
| 18 | 1.12 (s) | 16.4 | 18 | 1.16 (s) | 17.6 |
| 19 | 1.02 (s) | 16.5 | 19 | 0.96 (s) | 17.6 |
| 20 | - | 140.1 | 20 | - | 72.9 |
| 21 | 1.83 (s) | 13.1 | 21 | 1.40 (s) | 25.8 |
| 22 | 5.51 (t, 7.3 Hz) | 123.2 | 22 | - | 35.7 |
| 23 | - | 27.4 | 23 | - | 22.9 |
| 24 | 5.23 (t, 6.4 Hz) | 123.5 | 24 | - | 126.2 |
| 25 | - | 131.2 | 25 | - | 130.7 |
| 26 | 1.62 (s) | 25.7 | 26 | 1.66 (s) | 26.7 |
| 27 | 1.58 (s) | 17.7 | 27 | 1.63 (s) | 17.3 |
| 28 | 1.30 (s) | 28.8 | 28 | 2.11 (s) | 31.7 |
| 29 | 0.81 (s) | 15.7 | 29 | 1.47 (s) | 16.7 |
| 30 | 0.97 (s) | 16.9 | 30 | 0.79 (s) | 16.7 |
| 1′ | 4.95 (d, 7.4Hz) | 105.1 | 1′ | 4.96 (d,7.5 Hz) | 103.5 |
| 2′ | - | 83.4 | 2′ | - | 79.9 |
| 3′ | - | 78.3 | 3′ | - | 78.0 |
| 4′ | - | 71.5 | 4′ | - | 71.6 |
| 5′ | - | 77.9 | 5′ | - | 79.4 |
| 6′ | - | 62.5 | 6′ | - | 62.8 |
| 1′′ | 5.41 (d, 7.8 Hz) | 106.1 | 1′′ | 5.82 (d, 7.0 Hz) | 104.8 |
| 2′′ | - | 77.2 | 2′′ | - | 75.8 |
| 3′′ | - | 78.3 | 3′′ | - | 78.7 |
| 4′′ | - | 71.5 | 4′′ | - | 71.2 |
| 5′′ | - | 78.1 | 5′′ | - | 67.2 |
| 6′′ | - | 62.8 |  |  |  |

**Table S7** ^1^H and ^13^C NMR spectral data of **13**-**14** (600 MHz, C_5_D_5_N, *δ* in ppm, *J* in Hz).

|  | **13** |  |  | **14** |  |
| --- | --- | --- | --- | --- | --- |
| NO. | *δ*_H_ (*J* Hz) | *δ*_C_ | NO. | *δ*_H_ (*J* Hz) | *δ*_C_ |
| 1 | - | 39.5 | 1 | - | 39.6 |
| 2 | - | 27.7 | 2 | - | 26.7 |
| 3 | 3.51 (m) | 78.5 | 3 | 3.27 (dd, 4.6, 11.3 Hz) | 88.8 |
| 4 | - | 40.0 | 4 | - | 39.9 |
| 5 | - | 60.7 | 5 | - | 56.2 |
| 6 | 4.73 (m) | 74.1 | 6 | - | 18.3 |
| 7 | - | 46.0 | 7 | - | 35.8 |
| 8 | - | 39.2 | 8 | - | 39.9 |
| 9 | - | 50.4 | 9 | - | 50.1 |
| 10 | - | 41.1 | 10 | - | 36.8 |
| 11 | - | 32.1 | 11 | - | 30.9 |
| 12 | 3.95 (m) | 70.8 | 12 | - | 70.1 |
| 13 | - | 48.8 | 13 | - | 49.4 |
| 14 | - | 51.7 | 14 | - | 51.3 |
| 15 | - | 30.0 | 15 | - | 30.6 |
| 16 | - | 26.5 | 16 | - | 26.6 |
| 17 | - | 49.6 | 17 | - | 51.4 |
| 18 | - | 17.6 | 18 | 0.94 (s) | 16.2 |
| 19 | 1.26 (s) | 17.6 | 19 | 0.80 (s) | 15.9 |
| 20 | - | 72.9 | 20 | - | 83.4 |
| 21 | 1.38 (s) | 22.7 | 21 | 1.63 (s) | 22.5 |
| 22 | - | 43.2 | 22 | - | 32.8 |
| 23 | - | 22.5 | 23 | - | 25.3 |
| 24 | - | 126.0 | 24 | 4.72 (dd, 8.1, 4.3 Hz) | 89.9 |
| 25 | - | 130.7 | 25 | - | 146.0 |
| 26 | 1.70 (s) | 25.8 | 26 | 5.20 (br s), 5.04 (br s) | 113.3 |
| 27 | 1.65 (s) | 17.6 | 27 | 1.89 (s) | 17.6 |
| 28 | 2.17 (s) | 31.3 | 28 | 1.28 (s) | 28.0 |
| 29 | 1.39 (s) | 17.1 | 29 | 1.11 (s) | 16.5 |
| 30 | 0.99 (s) | 17.1 | 30 | 0.93 (s) | 17.2 |
| 1′ | 5.31 (d, 6.8 Hz) | 101.7 | 1′ | 4.95 (d, 7.3 Hz) | 105.1 |
| 2′ | - | 79.4 | 2′ | - | 83.2 |
| 3′ | - | 78.4 | 3′ | - | 78.3 |
| 4′ | - | 72.2 | 4′ | - | 71.5 |
| 5′ | - | 78.2 | 5′ | - | 78.1 |
| 6′ | - | 63.0 | 6′ | - | 62.8 |
| 1′′ | 6.49 (s) | 102.0 | 1′′ | 5.40 (d, 7.0 Hz) | 106.1 |
| 2′′ | - | 72.5 | 2′′ | - | 77.2 |
| 3′′ | - | 72.4 | 3′′ | - | 78.3 |
| 4′′ | - | 74.2 | 4′′ | - | 71.6 |
| 5′′ | - | 69.4 | 5′′ | - | 77.9 |
| 6′′ | 1.83 (d, 6.3 Hz) | 18.7 | 6′′ | - | 62.5 |
|  |  |  | 1′′′ | 5.22 (d, 7.0 Hz) | 98.2 |
|  |  |  | 2′′′ | - | 75.0 |
|  |  |  | 3′′′ | - | 79.2 |
|  |  |  | 4′′′ | - | 71.5 |
|  |  |  | 5′′′ | - | 77.9 |
|  |  |  | 6′′′ | - | 64.8 |

1. *Correspondence:

   Xiao-Yang Yang

   [yangxiaoyan9999@163.com](mailto:yangxiaoyan9999@163.com)

   Deng-Ji Lou

   [loudengji@yxnu.edu.cn](mailto:loudengji@yxnu.edu.cn)

   Full list of author information is available at the end of the article [↑](#footnote-ref-1)
